# Coordination and persistence of aggressive visual communication in Siamese fighting fish

**DOI:** 10.1016/j.celrep.2024.115208

**Published:** 2025-01-14

**Authors:** Claire P. Everett, Amy L. Norovich, Jessica E. Burke, Matthew R. Whiteway, Paula R. Villamayor, Pei-Yin Shih, Yuyang Zhu, Liam Paninski, Andres Bendesky

**Affiliations:** 1 Zuckerman Mind Brain Behavior Institute, Columbia University, New York, NY 10027, USA; 2 Department of Ecology, Evolution, and Environmental Biology, Columbia University, New York, NY 10027, USA; 3 Department of Statistics, Columbia University, New York, NY 10027, USA; 4 Lead contact

## Abstract

Outside acoustic communication, little is known about how animals coordinate social turn taking and how the brain drives engagement in these social interactions. Using Siamese fighting fish (*Betta splendens*), we discover dynamic visual features of an opponent and behavioral sequences that drive visually driven turn-taking aggressive behavior. Lesions of the telencephalon show that it is unnecessary for coordinating turn taking but is required for persistent participation in aggressive interactions. Circumscribed lesions of the caudal dorsomedial telencephalon (cDm; the fish pallial amygdala) recapitulated the telencephalic lesions. Furthermore, ventral telencephalic regions and the thalamic preglomerular complex, all of which project to cDm, show increased activity during aggressive interactions. Our work highlights how dynamic visual cues shape the rhythm of social interactions at multiple timescales. The results point to the vertebrate pallial amygdala as a region with an evolutionarily conserved role in regulating the persistence of emotional states, including those that promote engagement in social interactions.

## In brief

Everett et al. investigate the visual cues and neural substrates that drive turn taking and persistent aggression in Siamese fighting fish aggressive displays. They identify the pallial amygdala as essential for persistent aggression, highlighting its evolutionarily conserved role in regulating behavioral states across vertebrates.

## Graphical Abstract

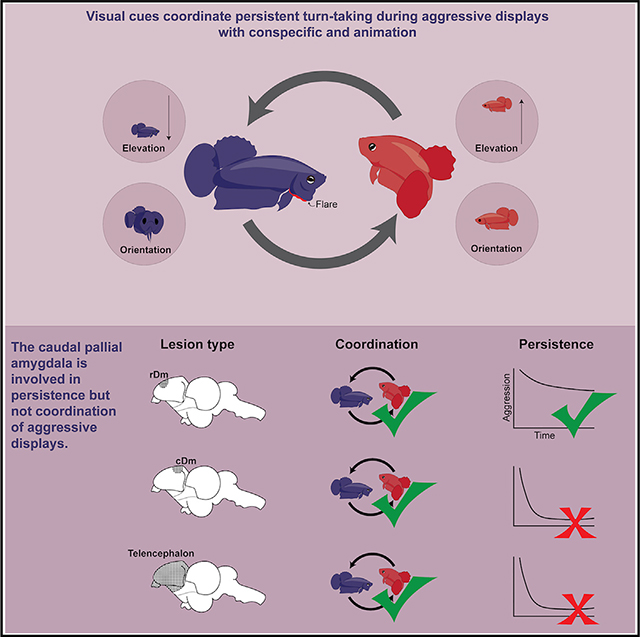

## INTRODUCTION

The coordination of behavior among individuals is a fundamental feature of social interactions.^1,2^ Such coordination can align the behaviors of two or more individuals in space and time, as seen during schooling in fish and flocking in birds, as well as during competitive encounters, such as head butting in rams.^3,4^ In other instances, animals anticorrelate their behavior, such that “turn taking” occurs. A canonical example of turn taking is acoustic communication, displayed by many invertebrate and vertebrate species in both cooperative and agonistic interactions.^5–7^ Turn taking in agonistic interactions often involves alternating between attack and defense, a pattern that Tinbergen referred to as “pendulum duels.” This can be seen in the ritualized exchange of strikes during territorial conflicts in mantis shrimp,^8,9^ as well as in the quick alternation between chasing and fleeing in sticklebacks^10^ and prairie horned larks.^11^

How animals determine when it is their turn to perform a behavior is best understood in acoustic interactions.^12,13^ In singing mice, for example, the end of a singing bout triggers the start of singing by a partner.^5^ During conversations, people predict the end of their interlocutor’s turn based on the lengthening of articulation^14^ and rapidly suppress speech if interrupted.^15^ This precise response timing is mediated by auditory neural circuitry that impinges on motor initiation circuits.^12^ The psychophysics, behavioral dynamics, and neuronal mechanisms of turn taking reliant on sensory modalities other than audition, however, are not well understood. Part of the difficulty in characterizing the precise sensory cues that shape turn-taking behaviors stems from the fast dynamics and highly correlated nature of multiple behavioral features, both within and across sensory modalities and within and across the interlocutors.

In addition to the neuronal circuits for timing turn taking, nodes involved in emotion and arousal likely influence whether individuals respond to their partner and hence shape the duration of interaction bouts.^12^ These emotion circuits appear to be distinct from the sensorimotor systems yet can exert significant influence over them.^16^ A core element of emotion circuits is the amygdala, an evolutionarily ancient structure present throughout vertebrates.^17^ In humans and other mammals, the basolateral amygdala (BLA) modulates affective states and promotes aggressive behaviors.^18,19^ In humans, the BLA is also important for detecting anger and other emotions in the faces of others.^19–21^ Whether the amygdala and other emotion circuit nodes promote continued participation in social interaction bouts, including those involving turn taking, or modulate the turn-taking dynamics has not yet been shown.

To study the timing and persistence of visually driven turn-taking behavior, we focus on the Siamese fighting fish (*Betta splendens*, or simply “betta”). Betta is a particularly aggressive species of freshwater fish endemic to Thailand that has been used for centuries in organized fights.^22,23^ Betta aggressive encounters start with a visual display phase, in which animals take turns at facing while flaring their opercular gill covers, alternating with turning sideways while spreading their fins and sometimes beating their tail.^24^ Although betta display behavior has not been studied in the wild, in controlled settings, the fish that flares less tends to surrender and flee.^24–27^ However, the display phase often escalates into an injurious phase involving biting.^24,25,28–30^ This suggests that the visual display can be considered a form of ritualized aggression during social encounters.

In this study, we dissect the dynamic interaction sequences and relevant visual cues through quantitative analyses of aggressive behavior of fish interacting with conspecifics or computer animations. We discover the visual features that shape turn-taking behavior acutely and those that promote continuous participation in the display phase. We then identify the brain regions that are particularly active while fish display aggression and that drive engagement in the aggressive display.

## RESULTS

### Betta take turns during aggressive displays

**Figure 1.**
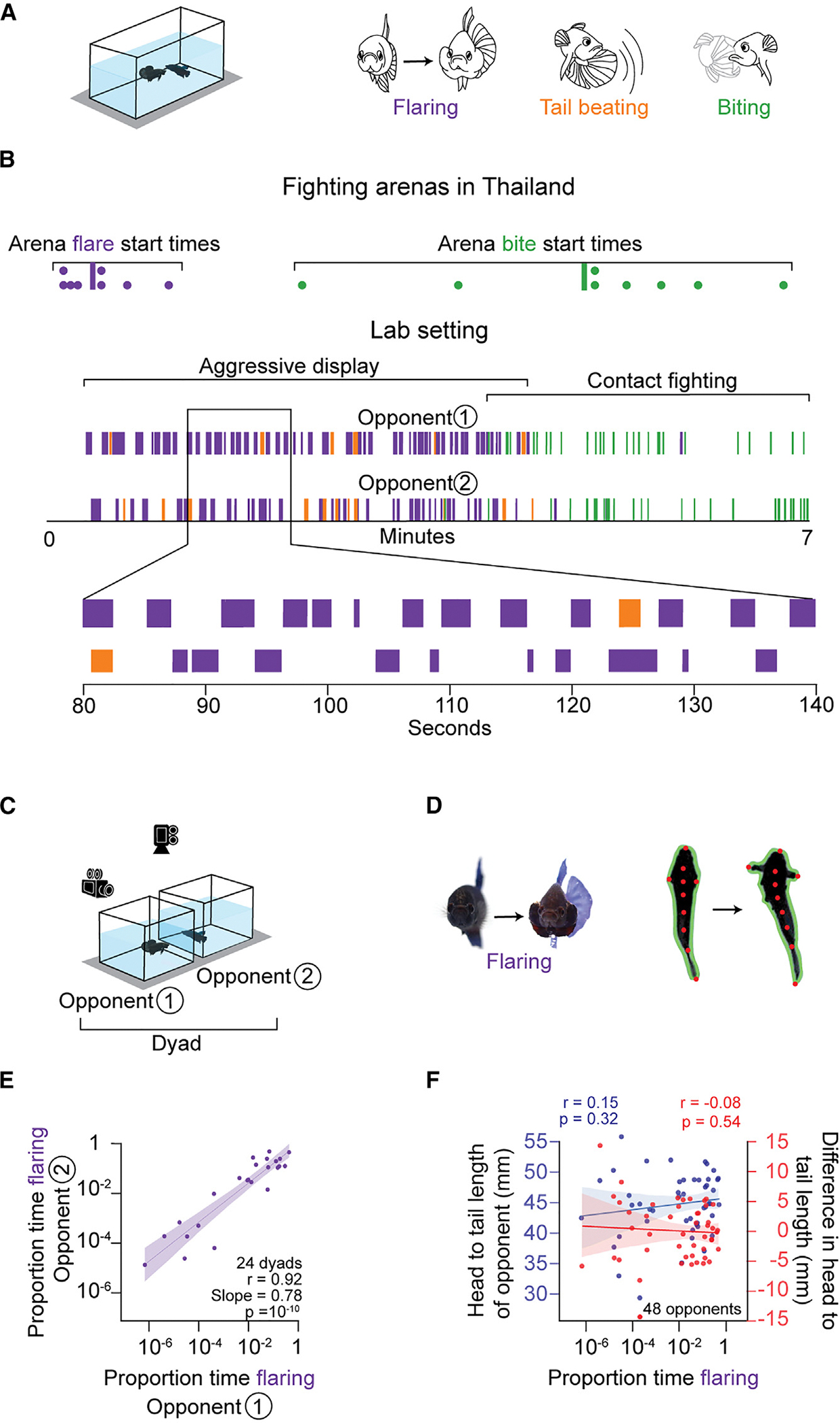
Betta take turns during aggressive displays and scale their aggressive response to match that of their opponent. (A) Opponents are placed in the same tank and multiple aggressive behaviors (flaring, tail beating, and biting) are scored. (B) Top: first flare and bite start times observed in fighting arenas in Thailand. Dots denote latency to first flare or bite in competitions between independent dyads, and vertical lines denote the mean. Bottom: ethogram raster plot of a single fight in a laboratory setting. (C) Behavioral paradigm consisting of a dyad in neighboring tanks. Videos were recorded from the top and side. (D) Fish key points (red dots) and contour (green outline) are tracked to quantify behavior. (E) Correlation of the proportion of time flaring between randomly chosen opponents 1 and 2. Each point denotes a dyad. (F) Correlation of the proportion of time flaring of either opponent 1 or 2 with the head-to-tail length of their respective opponent (blue) and the difference in head-to-tail length between opponents (red).

Turn-taking aggressive displays have been noted in betta located in adjacent transparent tanks.^24^ However, whether turn taking still occurs when individuals have physical access to each other (a more natural interaction) has not been documented. We placed a pair of male betta fish in the same tank and measured the flaring of the opercular gill covers (henceforth “flaring”), tail beating, and biting behaviors (Figures 1A and 1B). The first flaring event occurred 23 s after fish obtained physical access to each other, whereas the first tail beating occurred 34 s after the first flaring. Each flaring bout lasted an average of 1.1 s (±0.99 s standard deviation) and rarely overlapped with flaring by the opponent (*p* = 10^−48^ by Fisher’s exact test for lack of overlap; r= −0.94). During the first 4 min of the interaction, flaring occurred 18% of the time, and tail beating occurred much less frequently—only 2% of the time. The first bite was seen at 3.6 min into the interaction, and a clear transition into biting by both fish occurred only 30 s later. At that point, biting was the predominant behavior, while flaring and tail beating essentially disappeared. These laboratory results were consistent with our observations of betta fish fighting in organized contests in Thailand (Figure 1B). Across eight dyads, the first flaring event occurred, on average, 25 ± 16 s after fish were placed in the same tank. The first bite occurred, on average, at 4.95 ± 1.45 min. Thus, as in the laboratory setting, fish engaged in minutes-long aggressive displays before proceeding to the injurious biting phase. Altogether, these patterns are consistent with aggressive behavior in betta going through a display phase where animals take turns at flaring, followed by a contact-fighting phase where biting replaces flaring.

The contactless turn-taking aggressive displays of betta can be elicited by visual cues alone,^25,31–36^ and this is often achieved using a mirror.^34,35,37^ However, this mirror paradigm is not conducive to studying turn taking since fish see a simultaneous reflection of their own behavior. Instead, we examined turn-taking behavior by placing two fish in adjacent tanks while we video-recorded them from the top and side (Figure 1C). To automatically identify flaring behavior, we tracked the contour and multiple key points of each fish^38^ (Figure 1D). We then trained a supervised behavioral classification model^39^ to automatically score flaring behavior (also see STAR Methods). The model leveraged the tracked anatomical elements and the geometrical features derived from these elements, such as the angle formed between the tip of the nose and the tips of the operculum and the orientation of the fish, as well as basic movement behaviors, including speed and turning angle (Figure S1). This analytical procedure achieved an F1 score (reflecting both precision and recall using manual scoring as the ground truth) of 0.75 (Figure S1I). When we required even higher sensitivity and specificity, we complemented the automated method with manual scoring, and we note these cases in the STAR Methods.

Using these behavioral and analytical methods, we found that the fraction of time spent flaring by two randomly paired opponents across tanks was highly correlated (r = 0.92, *p* = 10^−10^; Figure 1E), consistent with previous findings.^24^ In contrast, the fraction of time spent flaring was not correlated with the absolute (r = 0.15, *p* = 0.32) or relative (r = −0.08, *p* = 0.58) size of an opponent (Figure 1F). These observations indicate that dynamic visual cues, rather than size (which modulates aggression in other fishes^40–42^), are sufficient for betta to scale their aggression to match that of their opponent.

### Two complementary paradigms to study visually evoked aggressive displays

**Figure 2.**
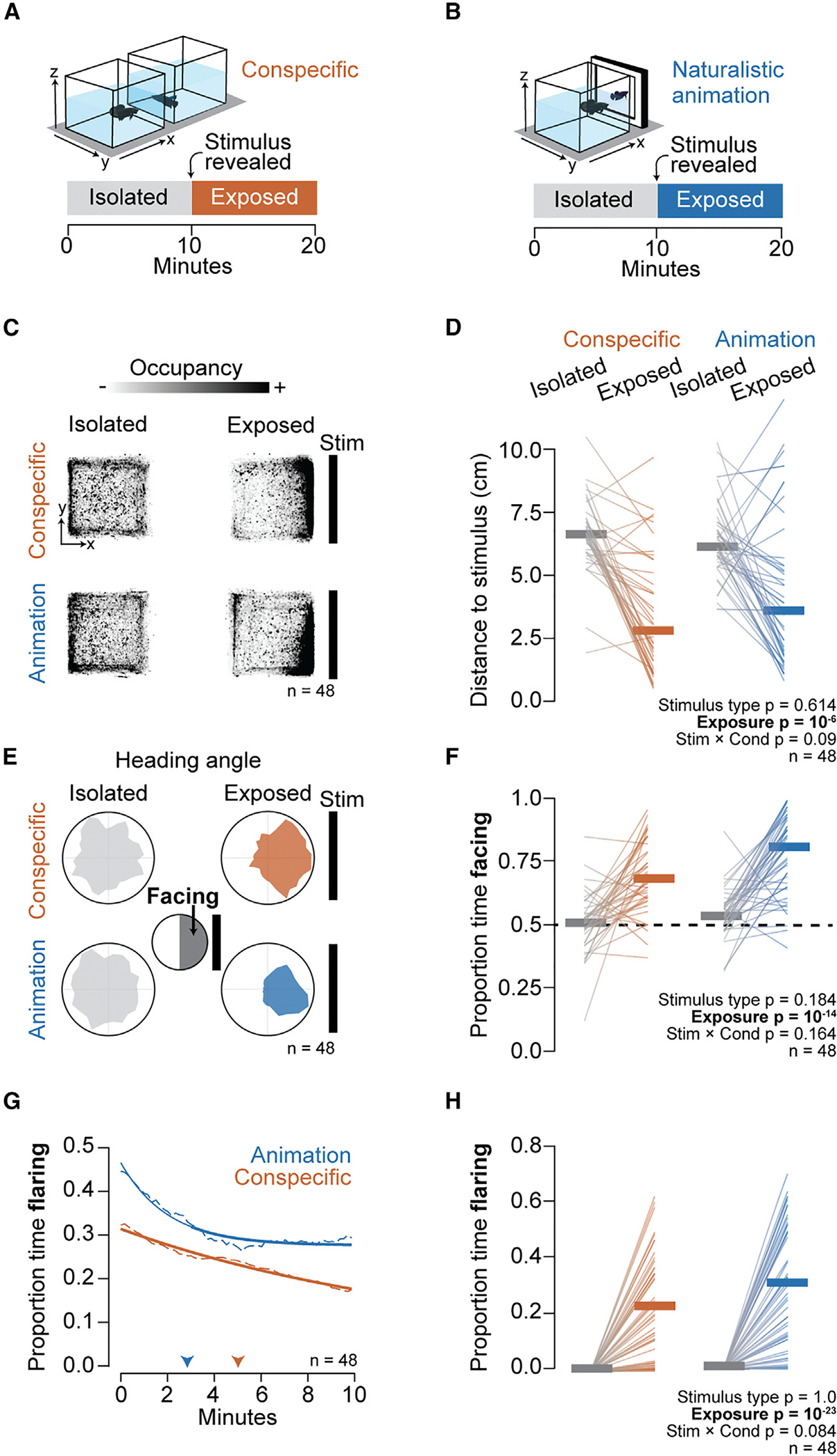
Naturalistic animations evoke as strong a flaring response as a conspecific. (A) Conspecific paradigm: two opponents in neighboring tanks. (B) Naturalistic animation paradigm: one opponent faces a naturalistic animation modeled after a male displaying aggression (see STAR Methods). (C) Heatmaps showing tank occupancy (from a top view) by fish during isolation and exposure. (D) Average distance to the stimulus. (E) Polar plots showing distributions of the head orientation during isolation and exposure. (F) Proportion of time spent facing within 180° of the stimulus. Dotted line denotes chance level. (G) Average persistence of the proportion of time flaring over the course of the exposure period (dashed lines) with exponential curve fit (solid lines). Arrowheads point to the time when the proportion of time flaring decreases to 0.75× of the max. (H) Proportion of time flaring during isolation and exposure. (D, F, and H) Thin lines denote individuals, and horizontal thick lines denote the median. *p* values were determined by mixed-model ANOVA.

To discover how visual cues shape turn-taking aggressive displays, we studied betta behavior in two complementary paradigms: (1) exposing individuals to another betta (a conspecific) in an adjacent transparent tank and (2) exposing individuals to a computer animation of a betta performing an aggressive display (Figures 2A and 2B; see STAR Methods). Each paradigm has strengths and weaknesses. The conspecific in an adjacent tank captures a palette of natural behaviors but is difficult to control because variability in behavior across time and individuals leads to a lack of consistency. Animations have the advantage that they can be presented repeatedly to individual fish and different individuals and that visual features can be precisely controlled; however, our animations do not respond to the observer fish, potentially affecting behavior. The animation was created by tracking a betta performing an aggressive display to a conspecific—with intervals of flaring as well as changes in orientation, velocity, and position— and then latching this real movement pattern onto the three-dimensional (3D) lattice of a realistic-looking betta (Videos S1, S2, and S3; STAR Methods). The animation is 37 s long and was shown for a total of 10 min (looped 16.2 times) to 17 animals. Fish are exposed to both paradigms in a random order, separated by a 10 min break. Prior to revealing the stimulus, animals spend only ~30% of the time in the quarter of the tank closest to the stimulus side (the other conspecific’s tank or the monitor) but ~60% of the time after reveal (*p* = 10^−9^). Consistent with this result, the average distance of the head of the fish to the side of the tank closest to the stimulus also decreased from ~7.5 to ~2.5 cm after reveal (*p* = 10^−4^; Figures 2C and 2D). Furthermore, in both paradigms, animals spend only ~50% of the time facing the stimulus side before reveal but ~68%–80% after reveal (*p* = 10^−14^; Figures 2E and 2F). We found no significant difference in time spent near the stimulus or time facing the stimulus between the real conspecific stimulus and the animation (stimulus × condition *p* = 0.67 and *p* = 0.18, respectively). In both paradigms, fish flared exclusively after the stimulus was revealed (exposure *p* = 10^−23^), consistent with flaring requiring visual cues. Upon stimulus reveal, fish flared less toward a conspecific than to the animation, but flaring against the animation decayed faster over time (Figure 2G). These flaring dynamics combined to yield a similar proportion of time spent flaring in both paradigms during the 10 min stimulus exposure period: ~22% against a conspecific and ~30% against the animation (stimulus × condition *p* = 0.08; Figure 2H). Differences in flaring decay might result from weaker habituation to a conspecific whose behavior varies through time than to an animation whose behavior repeats in a loop and from the lack of behavioral response of the animation to the real fish. Against both stimuli, as fish flared less over time, they also faced the stimulus less; however, fish remained engaged by staying near the stimulus across time (Figure S2). Altogether, the results indicate that fish engage with both types of stimuli to a similar extent.

### The visual cues that modulate betta aggressive behavior

**Figure 3.**
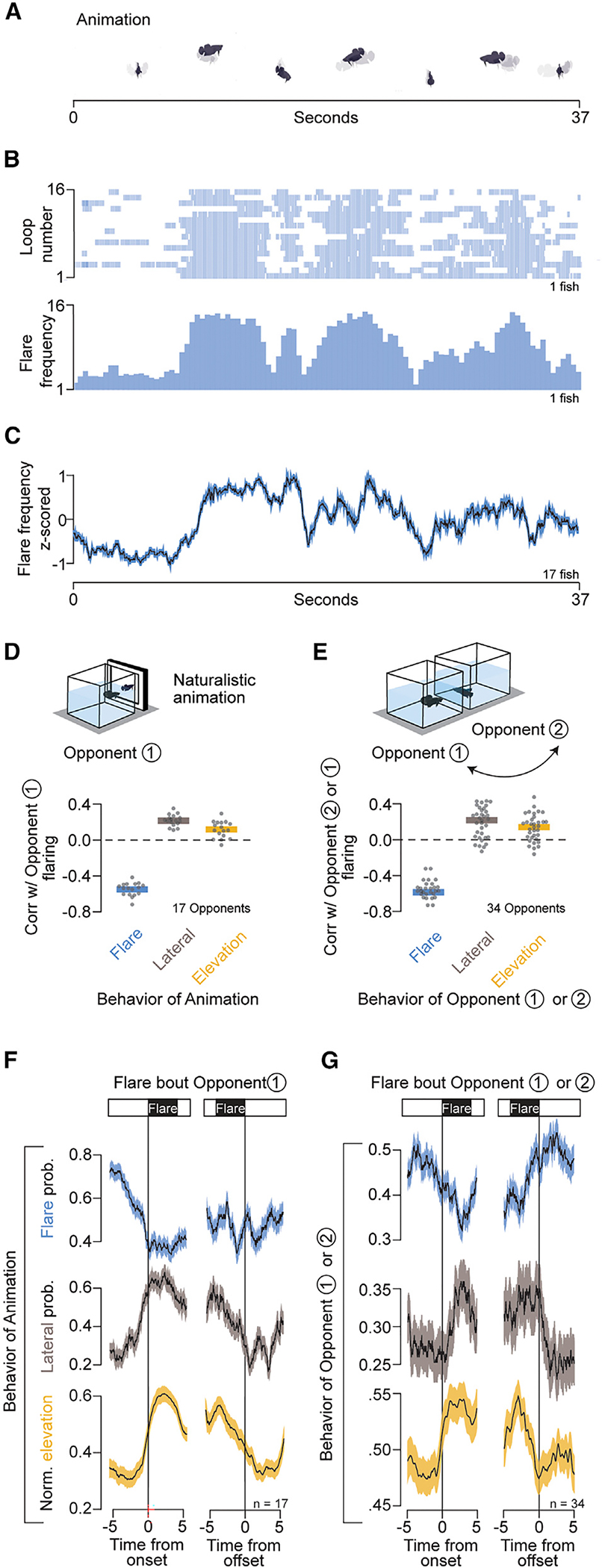
Betta coordinate their flare response with dynamic visual cues. (A) Selected frames from one loop of a naturalistic betta animation. (B) Raster plot (top) and histogram (bottom) showing flaring during each time point in the animation. (C) *Z-*scored flaring frequency (mean ± SEM). (D) Correlation between flaring of opponent 1 and behaviors of the animation. (E) Correlation between flaring of opponent 1 or 2 and behaviors of their opponent. (F and G) Peri-event time histograms (mean ± SEM) of changes in flare probability, lateral orientation probability, and normalized elevation (see STAR Methods) of the virtual fish (F) or real opponent (G) aligned to either the onset (left) or offset (right) of flaring bout of the opponent.

We took advantage of the repetitive nature of the animation (presentation in a loop 16.2 times) to determine how different epochs of the animation relate to flaring responses of the observer. Notably, fish synchronize their flaring behavior to defined intervals of the animation (Figures 3A and 3B). Qualitatively, fish flare the least when the animated fish is facing forward and most when the animated fish is lateral, and they demonstrate this pattern repeatedly across loops of the animation. Overall, 81% of fish (17/21) synchronized their flaring (see STAR Methods; Figure S3) across loops of the animation (Figure 3C). To quantitatively relate flaring behavior to specific features of the animation, we measured the correlation between flaring and multiple dynamic features of the animation. These features included position, speed, velocities in different directions, acceleration, and elevation, as well as orientation and flaring of the animated fish (Figure S4). The strongest correlations were with flaring (r = −0.55), lateral orientation (r = 0.3), and elevation (r = 0.2) (Figure 3D). The anticorrelation with flaring is consistent with the turn-taking behavior observed when two fish have physical access to each other (Figure 1B). Flaring occurs almost exclusively when betta face their opponent (Figures S5 and S6). Therefore, the correlation between flaring and a lateral orientation of the animated fish is also consistent with turn taking. The correlation between flaring and the elevation of the animated fish was surprising, and we address this in a later section. The correlations between the behavior of the animation and the flaring of the observer were remarkably consistent when measured against a real conspecific, indicating that the animation captured visual elements that naturally trigger a flaring response (Figures 3E and S4).

To parse the correlations between stimulus and flaring response in a more temporally precise manner, we generated peri-event time histograms aligned to the beginning or end of a flaring event (Figures 3F and 3G). This allowed us to characterize which behaviors, either of the animation or the real opponent, may cause flaring to start or stop. We found that fish began to flare when the animated fish stopped flaring, went lateral, and elevated itself. By contrast, fish stopped flaring as the animation turned from lateral to facing forward and as it descended on the screen. Interestingly, fish did not time the end of a flare with the start or end of a flare from the animation, suggesting that observing a change in orientation from lateral to facing and a decrease in elevation is sufficient to interrupt flaring. Interactions between real opponents led to qualitatively similar observations (Figure 3G), though interpretations are more difficult because interacting fish respond dynamically to each other, whereas fish cannot influence the behavior of the animation. Overall, three-fourths of flaring bouts start when the opponent has started to close its operculum or has stopped flaring altogether. In Figure S5, we present an analysis of behavioral transitions between both fish and the probability of each pathway leading to the onset and offset of flaring. The results from quantitative analysis of aggressive encounters with conspecifics and animations suggest that both the onset and offset of a flaring event are regulated by specific visual cues, that animals start flaring when their opponent turns lateral, stops flaring, and elevates itself, and that they stop flaring spontaneously or when their opponent turns to face them and moves downward in the water column.

### Role of flaring in the aggressive display

**Figure 4.**
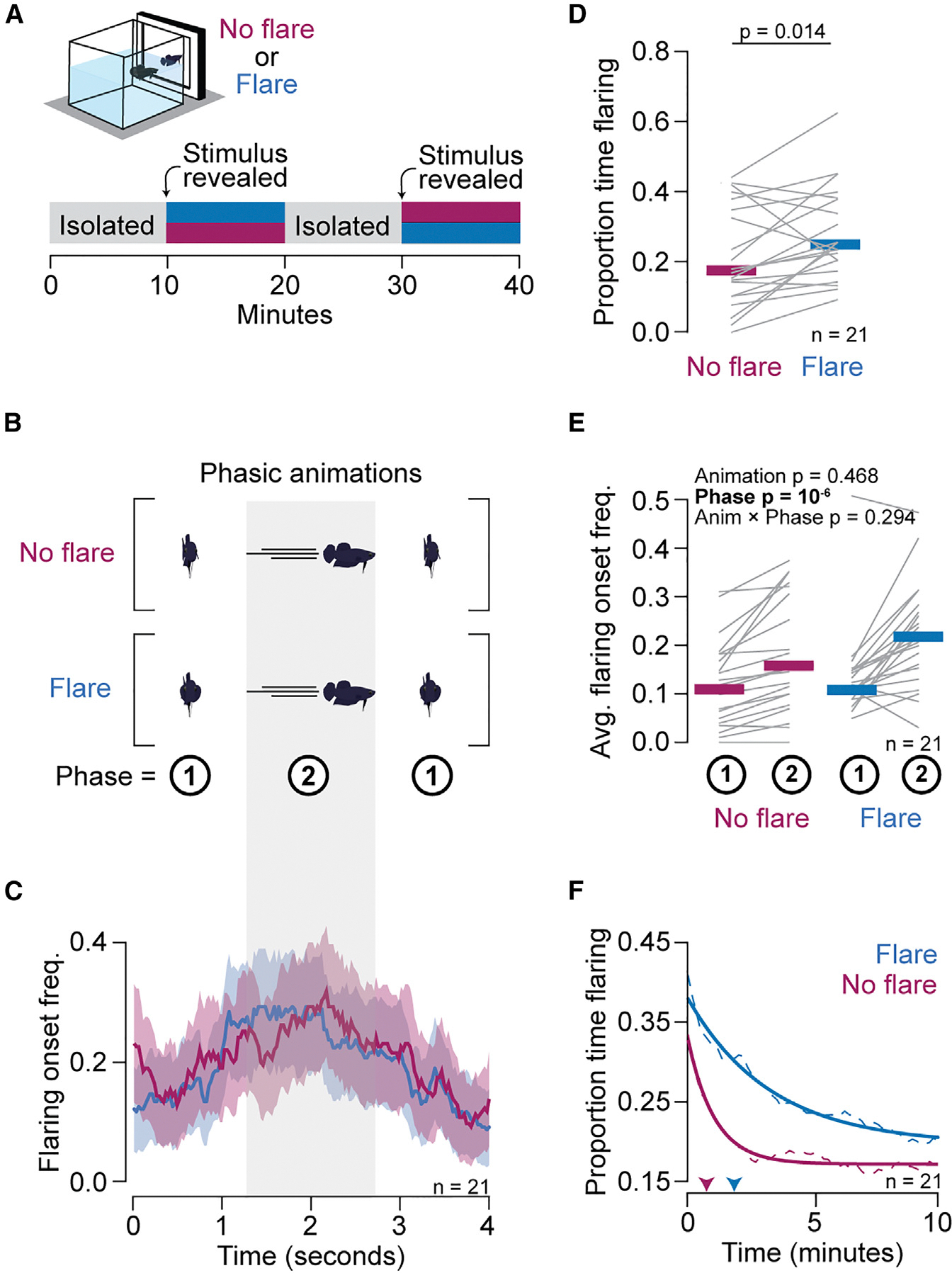
Flaring of a stimulus is not necessary for synchronizing flaring but promotes more persistent flare responses. (A) Behavior setup for presenting two phasic animation types (no flare or flare) to fish in a balanced order. (B) Animations were composed of two alternating phases with distinct combination of flare, orientation, and speed state. (C) Frequency of flaring bout onsets (mean ± SEM) aligned to a loop of the animation. (D) Proportion of time flaring against each animation. (E) Average flaring onset frequency for each phase (1 or 2) in each animation. (D and E) Thin gray lines denote individuals, and horizontal thick lines denote the median. *p* values were determined by a two-tailed paired t test (D) or mixed-methods ANOVA (E). (F) Average persistence of the proportion of time flaring over the course of the exposure period (dashed lines) with exponential curve fit (solid lines). Arrowheads point to the time when the proportion of time flaring decreases to 0.75× of the max.

As part of turn taking, betta initiate flaring around the time when their opponent stops flaring. However, the cessation of flaring coincides with other behavioral changes (Figure S6), making it difficult to determine which specific actions of the opponent trigger a flaring response in the observer. For example, around the end of a flaring bout, fish transiently increase their turning angle as they turn into a lateral orientation (Figure S6). To decouple flaring from facing, we created two animations in which a fish alternates between two phases, (1) facing forward while stationary and (2) turning lateral and swimming to the other side of the screen, in a loop (Figures 4A and 4B; Videos S4 and S5). The two animations differ only in whether fish flare while facing forward. Twenty-one individuals were exposed to both animations (each in a 10 min block) in a balanced order. The onset of flaring bouts reached its maximum when the animated fish turned lateral and its minimum when the animation faced forward (Figure 4C). Conversely, offsets of flare bouts reached a maximum when the animated fish faced forward (Figure S7). Notably, this pattern was indistinguishable regardless of whether the animated fish flared or not. Consistent with the time histograms (Figures 3F and 3G), a linear model for flaring onset was significantly affected by the phase of the animation (β = 0.08; *p* = 10^−6^) but not by the type of animation (flaring or not flaring; β = 0.008; *p* = 0.47), nor by an interaction between the animation phase and type (β = 0.025; *p* = 0.29; Figure 4E). Interestingly, although flaring by the stimulus did not acutely affect flaring by the observer, it did lead to a 41% (*p* = 0.014) increased median time flaring during the 10 min of the animation (Figure 4D). This increase in total flaring time can be partly attributed to more persistent flaring across the 10 min of exposure (Figure 4F). These results indicate that betta can modulate their flaring response from watching an opponent change in orientation between lateral and forward facing and that flaring is not required for turn-taking behavior. Instead, flaring seems to encourage continued engagement in aggressive interactions.

### Role of elevation in the aggressive display

**Figure 5.**
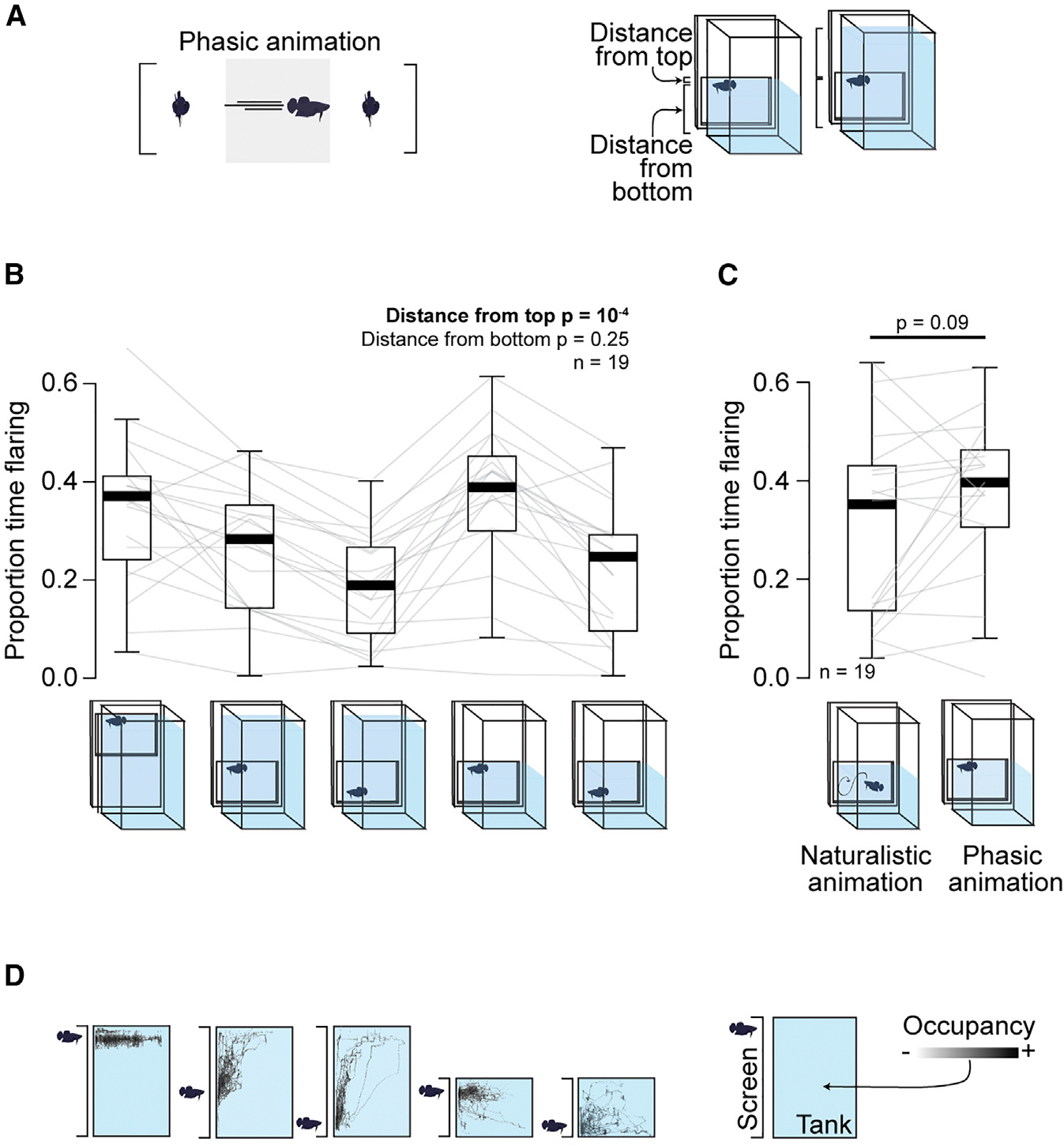
Flaring increases when the opponent is close to the surface. (A) Left: phasic animation in which the stimulus maintains its elevation. Right: animation was shown at different distances from the surface and bottom of water by varying the elevation of the animation and the height of the water. (B) Proportion of time flaring against stimuli with varying distances from surface and bottom. (C) Proportion of time flaring against the naturalistic animation versus the phasic animation at elevation closest to the surface. (B and C) Boxes denote interquartile range with whiskers at 1.5× the interquartile range and lines at the median. (D) Heatmaps of exemplar individual betta against each elevation condition.

Our finding that a higher elevation of the opponent promotes a flaring response (Figures 3D–3G) was unexpected, as this has not been described as a relevant visual cue for aggressive behavior in betta or other aquatic animals. Elevation in the water column, however, can have important ethological relevance since many elements of the life of betta occur near the surface. As anabantoids with a lung-like labyrinth organ, betta can breathe air.^43^ During fights, betta increase oxygen consumption and the rate of ascension to the water surface to breathe.^24,26,30^ Furthermore, betta males build bubble nests at the water surface and show aggressive territorial behavior near their nests.^24,43,44^ Therefore, we hypothesized that animals display more aggression (a sign of territoriality) the closer their opponent is to the surface rather than the farther it is from the bottom. To test this hypothesis, we exposed 19 fish to an animation presented at varying distances from the top and from the bottom, decoupling the two by also varying the height of the water (Figures 5A and 5B). Consistent with the results of encounters with real and animated opponents, in which fish flare more when the opponent is more elevated, fish facing these simpler animations that maintain their height flared the most to opponents that appeared closest to the surface and least to opponents closest to the floor (Figure 5B). A generalized linear model supported the hypothesis that distance from the surface is more relevant for triggering flaring than distance from the bottom (β = 0.073, *p* = 10^−4^ for distance from the top; β = 0.021, *p* = 0.25 for distance from the bottom). Notably, when presented at the top of the water column, the simple phasic animation that alternates between facing and flaring with turning laterally and moving to the other side (the “flare” condition in Figure 4) evokes as much flaring from the observer as the more complex naturalistic animation (Figure 5C). This attests to the strength of a simple stimulus in eliciting aggression, if presented close to the surface.

We further explored how betta adjust their own elevation to interact with stimuli at different depths. When presented with the animations that do not change in depth, animals aligned themselves with the stimuli (Figure 5D). However, this alignment was more variable the farther the stimuli were from the surface. This might reflect a stronger drive to interact aggressively with opponents near the surface, difficulty in maintaining the buoyancy necessary to go to the bottom (since betta take in air more frequently when they fight), or less time spent close to the bottom due to the frequent trips to the surface to breathe. In encounters between two real animals, before gaining visual access to each other, there was a bimodal distribution of elevation, with animals spending most of their time close to the bottom or close to the surface and the least time in between (Figure S8). After exposure to each other, however, both animals spent most of their time close to the surface (Figure S8), suggesting a preference for an elevated position during displays.

### The caudal dorsomedial telencephalon promotes engagement in aggression

**Figure 6.**
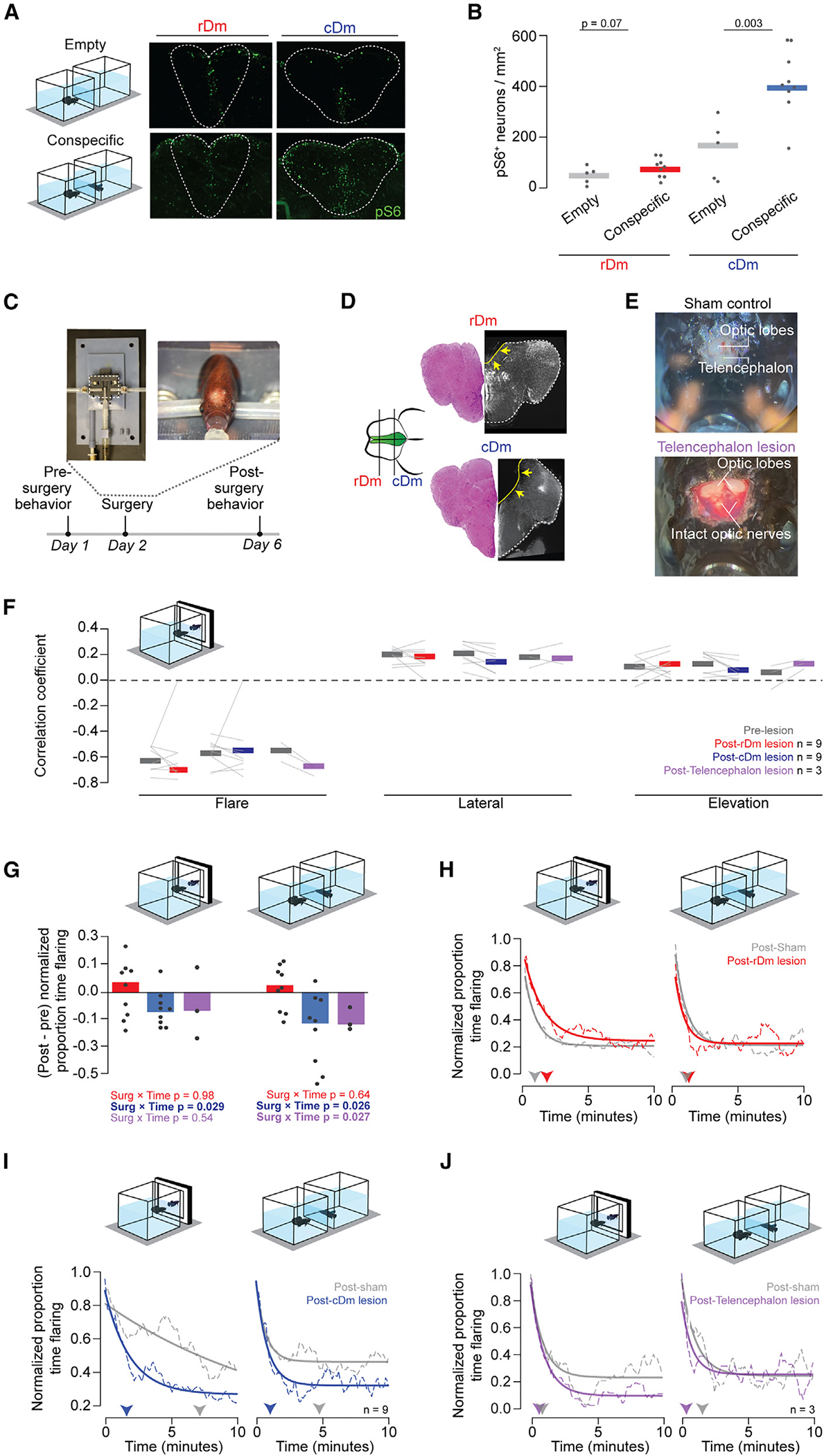
Caudal Dm promotes persistent engagement in an aggressive interaction. (A) Left: betta were exposed to either a conspecific or empty tank for a brain-wide activity screen. Right: example sections of rDm and cDm with pS6+ cells. (B) Quantification of pS6+ cells in rDm and cDm following conspecific or empty tank exposure. *p* values were determined by Welch’s t test. For other brain regions, see Figure S9. (C) Custom survival surgery set up for Dm lesioning and experimental timeline. (D) Example sections showing targeted lesions of rDm and cDm. Arrows point to the brain region removed by the lesion. (E) Example telencephalon removal with intact optic lobes and optic nerves following removal. (F) Correlations between flaring of fish with rDm (red), cDm (blue), or telencephalon (purple) lesions pre- and post-surgery with behaviors of the naturalistic animation. (G) Change in the proportion of time flaring toward animation (left) and conspecific (right) following lesions of rDm, cDm, or telencephalon, normalized by change in the proportion of time spent flaring of sham-operated individuals. *p* values were determined by mixed-methods ANOVA. (H–J) Normalized average persistence of time flaring over the course of the exposure (left: animation; right: conspecific) period (dashed lines) with exponential curve fit (solid lines), normalized to the maximum flaring of each individual. Arrowheads point to the time when the proportion of time flaring decreases to 0.5× of the max following sham or rDm lesions (H), sham or cDm lesions (I), and sham or telencephalon lesions (J).

Next, we aimed to determine which brain regions modulate the aggressive display. To that end, we began by performing a screen for neurons that were active when fish were engaged in an aggressive display (Figures 6A, 6B, and S9). We used phosphorylation of the ribosomal S6 protein (pS6) as a proxy for neuronal activity during display.^45,46^ We focused on 28 brain regions that could be defined anatomically using NeuroTrace labeling with reference to betta telencephalic atlases.^47,48^ This included the pallial and preoptic area subdivisions. We also quantified other forebrain regions, including the hypothalamus, habenula, preglomerular complex, torus longitudinalis (TL), and other areas that could be defined by anatomical homology to zebrafish^49^ and had visible pS6 labeling. Four out of the 28 brain regions had a significant (*p* < 0.05) increase in the number of pS6^+^ neurons in displaying animals compared to unexposed controls (Figure S9). No region showed a decrease in activity during display. The four regions with increased activity were the caudal preglomerular nucleus (PGc; 3.1× increase in pS6+ cells, *p* = 0.017), the supracommissural (1.5×, *p* = 0.026) and postcommisural (2.3×, *p* = 0.029) nuclei of the ventral telencephalon (Vs and Vp, respectively), and the caudal dorsomedial telencephalon (cDm; 2.3× *p* = 0.003). PGc is known to represent social signals including the identity of conspecifics in weekly electric fish.^50^ The ventral telencephalon promotes engagement in social interactions in zebrafish.^51^ cDm has previously been suggested to promote aggression in betta^29^ and arousal and defensive behaviors in several other teleost fishes.^52–54^ Based on its development and cytoarchitecture, Dm is considered homologous to the tetrapod pallial amygdala.^55–58^ Thus, these four regions that are active while betta engage in aggressive displays are known to regulate social behaviors across fishes.

Facilitated by its accessibility at the dorsal surface of the brain, we examined the role of cDm in aggression by lesioning it and subjecting the lesioned animals to our controlled behavioral assays. A previous study lesioned cDm in betta, but it did not report the extent or accuracy of the lesions or any controls.^29^ We bilaterally ablated this region by aspiration using a novel setup for betta stereotaxic surgeries (Figures 6C, 6D, and S10). To control for a potential effect of the surgery on aggressive display behavior, we sham lesioned animals by putting them through the same surgical procedure, including anesthesia and craniotomy, but without lesioning their brain. As an additional comparison to cDm lesions, we also lesioned rostral Dm (rDm), which did not show a significant increase in pS6 labeling following aggressive displays (1.1× relative to unexposed fish, *p* = 0.07; Figure 6B). The behavior of experimental animals was quantified before and after surgery to enable more powerful within-subject comparisons. We let animals recover for 4 days after surgery, minimizing the opportunity for plasticity post-lesion, which can complicate the interpretation of the effect of lesions.^59^

Compared to controls, lesions did not significantly alter swimming speed, distance to the stimulus or the proportion of time spent facing it, or the number and duration of flaring bouts (Figures S11 and S12). We next evaluated whether cDm or rDm might be involved in the synchronization of displays with the naturalistic animation. Neither cDm nor rDm lesions affected the timing of flaring relative to the animation (Figures 6F and S13). However, cDm lesions, but not rDm lesions, reduced the proportion of time spent flaring toward both the naturalistic animation and a real opponent (Figure 6G). This reduced flaring of cDm-lesioned animals was mostly a consequence of decreasing participation in the aggressive display over time: animals with cDm lesions began flaring at similar levels as sham-lesioned animals, but their flaring rate decayed faster over the next 10 min (Figure 6I). In contrast, rDm lesions did not affect the trajectory of flaring rates compared to sham-lesioned controls (Figure 6H). Thus, cDm is necessary for promoting continued engagement in aggressive interactions, a manifestation of a long-lasting emotional state.

In zebrafish, ventral telencephalic lesions disrupt the orientation of animals toward a conspecific visual stimulus.^51^ However, these lesions also reduce the time animals spend near the stimulus, indicating that they are overall less engaged in social interactions, making it difficult to assess the specificity of the orientation deficits. Still, we wanted to test whether the ventral telencephalon could also be important for betta orientation and turn-taking behavior. It is challenging to lesion ventral parts of the brain with the same precision as our dorsal lesions. Therefore, we asked whether the telencephalon as a whole is required for orientation and turn-taking aggressive displays in betta. Notably, we found that animals in which we removed the whole telencephalon do not spend less time close to the stimulus (a conspecific or the animation) or oriented away from it (Figure S14). Lesioned animals still synchronized their flaring behavior with the animation just as controls: flaring was inversely correlated with flaring by the animation and positively correlated with the animated fish being lateral and elevated (Figure 6F). However, as with cDm lesions, animals without telencephalon spent less time flaring, and the rate of decay of flaring was faster than control animals with mock lesions (Figures 6G and 6J). The telencephalic lesions spare PGc (which is also active during betta aggressive interactions), suggesting that PGc is not sufficient for driving persistent flaring behavior. Altogether, these results indicate that the telencephalon is dispensable for the sensorimotor transformations involved in turn-taking behavior and for approaching and orienting toward a stimulus but is required for maintaining robust flaring behavior. The effects of the telencephalic lesions were not more severe than the cDm lesions. Thus, whereas additional regions might also be involved in modulating emotional states conducive to participating in aggressive interactions, the results point to a prominent role of cDm.

### Overlapping and unique cDm and rDm afferents

**Figure 7.**
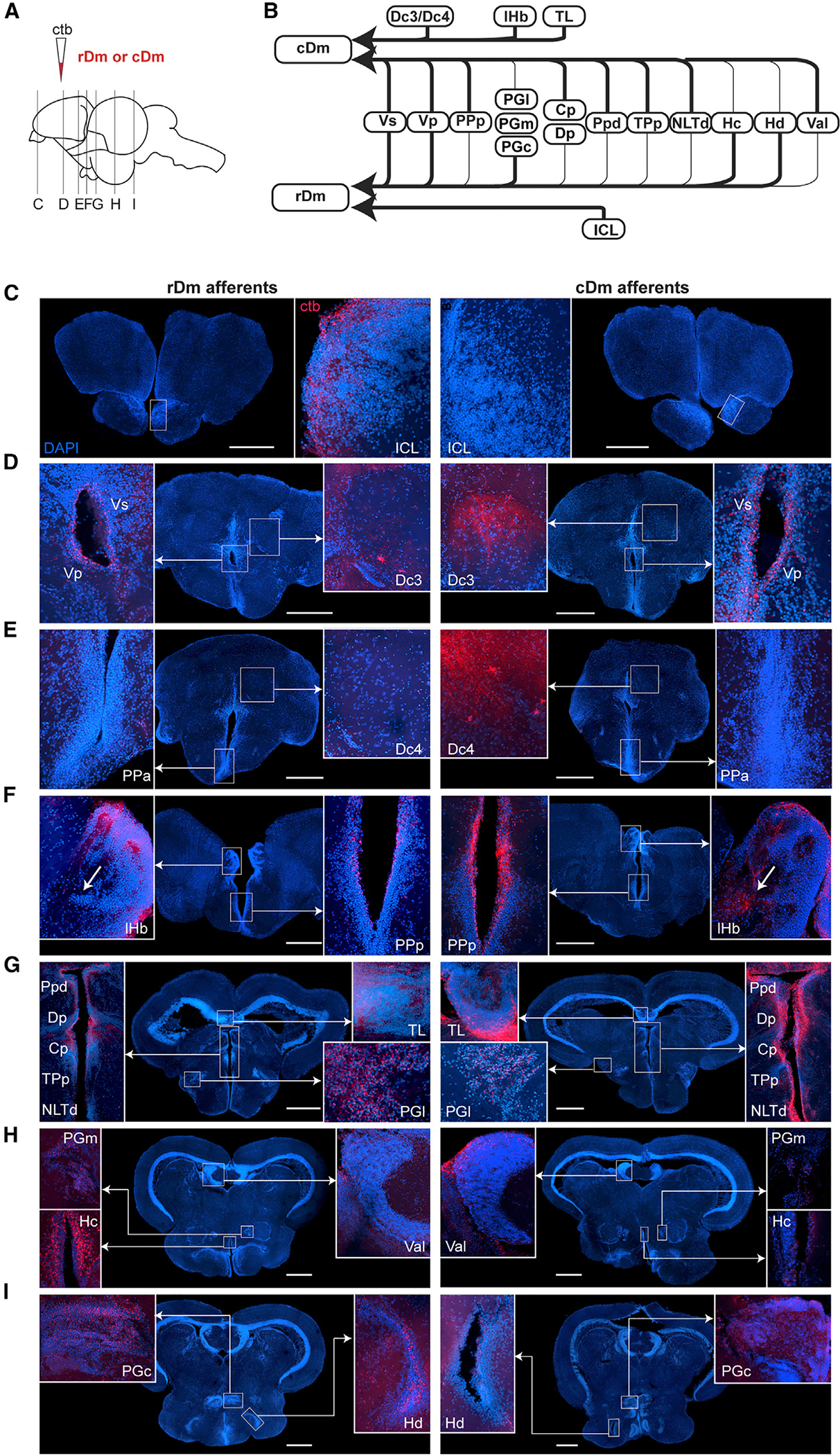
Neuronal projections to rDm and cDm. (A) ctb-555 was injected into either rDm (*n* = 3) or cDm (*n* = 3). (B) Diagram of afferents to rostral and caudal Dm. All ctb+ regions were consistently found in all three fish injected in each region. (C–I) Coronal sections of fish injected into rDm (left) or cDm (right) ordered along rostro-caudal axis with areas of interest highlighted. All highlighted areas were found in 3/3 rDm- or cDm-injected fish. Cp, central posterior thalamic nucleus; Dc3/4, central part of the dorsal telencephalon, subdivision 3/4; Dp, dorsal posterior thalamic nucleus; Hc, caudal zone of periventricular hypothalamus; Hd, dorsal zone of periventricular hypothalamus; ICL, internal cellular layer of the olfactory bulb; lHb, lateral habenula; NLTd, lateral tuberal nucleus, dorsal part; PGc, caudal preglomerular nucleus; PGl, lateral preglomerular nucleus; PGm, medial preglomerular nucleus; PPa, parvocellular preoptic nucleus, anterior part; PPp, parvocellular preoptic nucleus, posterior part; Ppd, dorsal periventricular pretectal nucleus; TL, torus longitudinalis; TPp, periventricular nucleus of the posterior tuberculum; Val, lateral division of valvula cerebelli; Vp, postcommissural nucleus of the ventral telencephalon; Vs, supracommissural nucleus of the ventral telencephalon. Nomenclature was informed by betta,^47^ cichlid,^62^ and zebrafish^49^ atlases. Scale bars: 400 μm.

We aimed to determine if the contrasting roles of rDm versus cDm in visually guided aggression were reflected in their connection patterns. To investigate whether rDm and cDm receive unique or overlapping afferents, including from the visual system, we conducted retrograde tracing using cholera toxin β (ctb). We found both shared and divergent inputs to these two areas (Figure 7). Consistent with observations in other teleost species,^56,60,61^ both rDm and cDm received input from ventral telencephalic regions (Vs and Vp), posterior parvocellular preoptic nucleus (PPp), preglomerular nuclei (lateral PG [PGl], medial PG [PGm], and PGc), thalamic nuclei (dorsal posterior thalamic nucleus [Dp] and central posterior thalamic nucleus [Cp]), and hypothalamic nuclei (caudal zone of periventricular hypothalamus [Hc] and dorsal zone of periventricular hypothalamus [Hd]). Interestingly, increased neuronal activity following exposure to a conspecific male—as shown in our brain-wide activity screen (Figure S9)—was observed in subpallial (Vs and Vp) and preglomerular nuclei (PGc), which project to Dm. This suggests the existence of a putative subpallial and preglomerular → pallial circuit at the core of visually evoked aggression in betta fish.

There were multiple unique afferents to rDm and cDm. Only rDm received projections from the olfactory bulb (internal cellular layer area [ICL]). In contrast, only cDm had projections from the dorsal central area of the pallium (Dc3/Dc4), the lateral habenula (lHb), and TL. Observing ctb+ cell bodies in TL in cDm-injected fish was surprising, since, to our knowledge, TL has not previously been shown to send projections to any region of the telencephalon in teleosts. This is a notable finding since TL receives visual input indirectly from the tectum and visual pretectum.^63,64^ TL forms a major reciprocal circuit with the tectum, which has been studied in several fish species,^63–67^ and the circuit has been theorized to be critical for visual attention and shape discrimination in goldfish.^68,69^ cDm might also affect participation in aggressive interactions irrespective of visual input (i.e., by relaying an aggressive behavioral state or a more general state such as arousal), but it is possible that visual input is important. To test whether cDm or rDm receives direct visual input from the eye, we performed anterograde tracing from the retina using ctb, which is transported anterogradely in retinal ganglion cells (RGCs).^70–72^ Consistent with findings in other teleost fishes,^73,74^ we observed ctb+ RGC terminals in the optic tectum and in a cluster of pretectal nuclei but not in cDm or rDm (Figure S15). Altogether, our results suggest that cDm receives inputs from preglomerular and other thalamic nuclei that could convey visual information via a conserved visual pathway.^61^ Alternatively, cDm may receive visual information through a novel pathway via TL.

## DISCUSSION

By studying visual encounters of betta with conspecifics and controlled animations, we discover key behavioral features that shape turn-taking aggressive behavior. When fish 1 stops flaring and facing and begins to reorient laterally, this triggers facing and flaring by fish 2; fish 2 then stops flaring and turns lateral, inducing facing and flaring by fish 1, leading to turn-taking cycles. Sometimes fish do not wait for “their turn” and instead start facing their opponent and flaring while the opponent is still flaring, which induces the opponent to stop flaring and turn sideways. This synchronization with occasional interruptions resembles the turn taking of facial expressions in chimps^75^ and vocal communication in humans and other animals.^7,12,15,76–78^ These parallels in the behavioral dynamics of visual and acoustic communication suggest that similar neuronal circuit logic gates their respective sensorimotor transformations.^12^

Because fish flare almost exclusively when facing their opponent, flaring and orientation are highly correlated. By presenting fish with animations that decouple these behaviors, we find that facing forward without flaring is enough to suppress flaring from the opponent. Notably, although flaring is not required to acutely modulate flaring by the opponent, it promotes continued engagement in aggressive display by the opponent over a timescale of minutes. Therefore, some visual cues shape social interactions acutely, and others do so chronically.

We find that cDm is more active while animals display aggression. cDm does not appear to be involved in the sensorimotor transformations required for turn taking. Instead, cDm promotes continuous engagement in an aggressive display with an opponent. Dm shares developmental, cytoarchitectonic, and connectivity features with the tetrapod pallial amygdala, which corresponds to the BLA of mammals.^54–58,79,80^ Our findings indicate that in addition to developmental and anatomical homology, fish cDm, like the mammalian amygdala, is also involved in maintaining persistent behavioral states related to emotion and self-preservation, such as aggressive encounters.^81,82^ Thus, the amygdala appears to have an evolutionarily conserved role across vertebrates in regulating behavioral states.

In addition to cDm, the subpallial regions Vs and Vp and the diencephalic region PGc were also active while fish engaged in aggressive displays. Vs has homology to the tetrapod medial amygdala,^83,84^ which regulates aggression and other social behaviors,^85,86^ and PGc is a thalamic-like region that sends sensory, motor, and social information to the pallium.^50^ Not only are cDm, Vs, Vp, and PGc all active during aggressive encounters, but Vs, Vp, and PGc also project to cDm. This co-activity and connectivity indicate that Vs, Vp, and cDm form part of a network active during aggressive interactions. Another region for future investigation in persistent aggression in betta is the hypothalamus. We did not find evidence of increased activity in the hypothalamus during aggression but did find projections from the hypothalamus to cDm. Electrical stimulation of the cichlid hypothalamus triggers attacks.^87^ In mice, the ventromedial hypothalamus is a central hub for regulating aggression.^88^ These results underscore the deep homology in the neural circuitry underlying aggression across vertebrates,^89^ pointing to a network of brain regions including the pallial-BLA, subpallial-medial amygdala, and hypothalamic regions. It will be interesting to dissect how this network interacts with the circuits performing the sensorimotor transformations underlying visual coordination to shape the duration of dynamic social encounters fundamental to survival.

### Limitations of the study

We found strong evidence that cDm is important for promoting persistent engagement in aggressive interactions: (1) cDm is active while fish engage in aggressive displays, (2) lesioning cDm (but not adjacent rDm) reduces continued participation in aggressive displays, and (3) removing the whole telencephalon (which includes cDm) does not lead to more severe aggression deficits than lesioning just cDm. Future studies that manipulate genetically defined Dm neuronal populations either in betta^90^ or using transgenic lines in zebrafish^91^ will provide deeper insights into how Dm regulates aggressive behavior.

We found two additional telencephalic areas (Vs and Vp) and one diencephalic area (PGc) that were also significantly active while fish displayed aggression, and we did not yet test directly if their lesions affect turn-taking synchronization or persistence. Whereas the telencephalic lesions suggest that Vs and Vp do not have effects additional to cDm on promoting persistence in aggression, it is possible that they are also required. At the same time, the telencephalic lesions—which spare PGc—suggest that PGc is not sufficient for maintaining a long-lasting aggressive state.

Another limitation of our work is that to place cDm within brain neuronal circuits, we focused on finding its afferents. This revealed important insights, including a pathway by which visual information can reach cDm via TL. This TL→cDm connection could be verified using anterograde tracing from the TL. It will also be important to identify the afferents of cDm to more fully understand how it modulates aggression.

Finally, while our animations look and behave naturalistically, they do not respond to the behavior of the real fish. This property has advantages but also the limitation that animals may change their behavior if their partner is not responding to them. Our results found strong concordance between the behaviors of fish in interactions with real opponents and with the animation, but closed-loop animations will be useful to further probe betta behavior.

## RESOURCE AVAILABILITY

### Lead contact

Further information and requests for resources should be directed to and will be fulfilled by the lead contact, Andres Bendesky (a.bendesky@columbia.edu).

### Materials availability

This study did not generate any new reagents.

### Data and code availability

- Behavioral videos, manual scoring, feature files, animations, and 3D-printing design files can be found in the supplemental information or have been deposited at Dryad and are publicly available at https://doi.org/10.5061/dryad.7wm37pw2w as of the date of publication.
- All original code is in the supplemental information and has been deposited at Dryad and is publicly available at https://doi.org/10.5061/dryad.7wm37pw2w as of the date of publication.
- Any additional information required to reanalyze the data reported in this paper is available from the lead contact upon request.

## STAR★METHODS

### EXPERIMENTAL MODEL AND STUDY PARTICIPANT DETAILS

Adult male betta bred for fighting were used for all experiments including behavior. Fighting males were sourced from an independent breeder in Bangkok, Thailand, or bred in lab from those Thai fish. Ornamental males were only used for pS6 activity mapping and were either obtained from pet stores or bred in lab. All males were 6–18 months of age when tested. We found no correlation between the age at testing and flare rate (r = 0.07, *p* = 0.63). Betta were maintained under standard husbandry and housing conditions.^92^ Adult betta were individually housed and visually isolated from other fish for a minimum of two months before they were tested. At time of testing, animals were randomly assigned to control and experimental groups. All animal work was approved by the Columbia University Animal Care and Use Committee.

### METHOD DETAILS

#### Behavior

##### Behavior arenas

Enclosed behavioral arenas were constructed to ensure interactions with conspecifics and animations took place in a controlled environment devoid of visual disruptions. Arenas were constructed using 80/20 metal framing and white featureless foamboard walls. Each arena was 54 × 43×63 (height) cm. Waterproof string white LED lighting (7000k color temperature; SUPERNIGHT) lining the top of the walls was used to illuminate the arena. The room that housed behavior arenas was kept at 26°C. Each behavior arena had top and side Raspberry Pi 3 Model B+ cameras with Raspberry Pi NoIR Camera Module V2 lenses. Cameras ran at 40 frames per second at a resolution of 1280 × 720 pixels. Tanks sat on platforms latched into laser cut stands to ensure a uniform distance of 1 inch between tanks or between tank and monitor.

##### Conspecific encounters

Individuals were placed inside a cubic tank (5 × 5 × 5 inches) and water was brought to a height of ~4.5 inches. Tanks were separated by two Polymer Dispersed Liquid Crystal (PDLC) plates (12 × 6 inches, SW Store), similar to what has been used previously.^51^ These screens remain opaque during the habituation phase. At testing phase onset, current from Raspberry Pis is run through the PDLC screens causing them to become immediately transparent.

Fish were habituated in visual isolation from their opponent for 20 min. The last 10 min of habituation period were used to assess behavior during isolation. The PDLC screen then became transparent, and fish were given visual access to each other for a 10-min test period.

##### Animation encounters

Individuals were placed in a testing tank across from a monitor (4.6 × 7 inches LCD; ToGuard) controlled by a Raspberry Pi, displaying a white screen. At testing phase onset, the animations began playing on the monitor. Betta were habituated for 20 min followed by 10 min of exposure to the animated stimuli. If multiple animations were displayed to a fish, they were separated by 10-min breaks in which the monitor displayed a white screen.

When testing the role of distance to the surface versus bottom, a single fish faced eight animations (phasic animation at five different heights and naturalistic animation at three different heights). To avoid fatigue, animations were grouped into three different “clusters”. Each cluster contained 2–3 animations. Clusters were separated by 1-h breaks. Both the order of the clusters and animations within the clusters were randomized so that our results were not biased by potential priming or fatigue.

#### Designing animations

Animations were made using Blender v2.91. Naturalistic animations were crafted in two parts: 1) designing the 3D fish and 2) importing a naturalistic trajectory. The Blender file is provided as Data S1.

##### 3D fish

The volume and fin shape and size of the virtual fish were modeled based on the body shape and size of a representative male. The color was uniform across all parts of the fish (except the eyes, which were black) and was the average color value of a representative fighting male.

##### Trajectories

Naturalistic trajectories were created by tracking a male betta during an aggressive display using the motion tracking function in Blender (Video S1). The 3D-fish model was then latched to the track and the orientation and gill flaring were manually added.

Phasic trajectories were crafted using custom Python scripts and imported into Blender. The speed of the artificial paths (~2 cm/s) matches the average speed of 10 fish during their test periods in which they displayed toward visual stimuli.

##### Animation gill flare

The size of the operculum was changed manually. To optimize the ethological relevance of flaring state in animations we measured change in width of a facing fish when the operculum was closed vs. open in a population of fish (*n* = 24). We then took the average change in width (1.3 cm) and used that to inform the full extension of the operculum in the animated fish.

#### Histology, tracing, and ablation methods

##### Activity mapping

Betta were exposed to either a conspecific or empty tank for 30 min. Fish were euthanized 90 min after the end of exposure. Fish were euthanized by submerging in ice water until immobile, followed by rapid decapitation. Heads were collected and fixed overnight in 4% paraformaldehyde in 0.1 M phosphate buffer (PB) at 4°C. Following fixation, brains were dissected out and washed 3× in phosphate buffer saline (PBS) before dehydration in 30% sucrose in 0.1 M PB. Brains were embedded in OCT (Tissue Tek) for cryosectioning. 20 μm tissue sections were incubated overnight at 4°C with primary antibodies diluted in 0.2% Triton X-100 PBS solution (0.2% PBT). Following 3× PBS washes, sections were incubated with secondary antibodies diluted in 0.2% PBT for 1 h at room temperature. NeuroTrace labeling (ThermoFisher; 1:200 dilution, 20 min) was performed following incubation in secondary antibodies. Slides were covered using VECTASHIELD mounting medium with DAPI (Vector Laboratories).

Rabbit anti-pS6 (1:10,000; ThermoFisher, 44–923G, Lot #2066361) was used in this study. Secondary antibodies were generated in donkey and conjugated to Cy3 (1:1,000; Jackson Immunoresearch Laboratories).

Brain regions were identified based on NeuroTrace labeling by reference to published betta brain atlases.^47,48^ pS6+ neurons in each region were counted manually using the Cell Counter feature in FIJI2.^93^ Cell counts were normalized by the area of the ROI measured using the Area function in FIJI2.

##### Surgical platform design

The survival surgery platform was adapted from a 3D-printable mouse stereotaxic platform^94^ using Blender (Data S3,S4). Modifications included resizing the platform and adjusting specific features. A custom holding well was designed to securely accommodate the curved underside of the fish. An additional supporting holder was added to stabilize the sides of the body. Head bars made of disposable transfer pipette tips were secured on either side of the head. The platform was angled to align the skull of the fish parallel to the stereotaxic platform for surgery.

##### rDm, cDm, and telencephalon lesion

Prior to surgery, a pulled capillary needle (3.5 inches, Drummond # 3-000-203-G/X; Heat:480, Pull:30, Vel.:80, Time:150, Pressure:200; Sutter P-1000 micropipette puller) was painted with DiI (Invitrogen, D3911, 2–3 crystals dissolved in 1 mL ethanol), attached to a vacuum, and mounted to a stereotaxic arm. Fish were then anesthetized by submersion in tricaine methanesulfonate stock solution (MS-222; Syndel, 0.08%, pH 7–7.5 using Tris buffer pH 10) diluted 1:10 in water optimized for betta husbandry.^92^ Fish were intubated by inserting the tip of a transfer pipette attached to tubing into the mouth to flow tricaine over the gills during surgery. Anesthesia during surgery was a 1:50 dilution of MS-222 stock in betta water and flowed at a rate of 1 mL/min. Scales and cutaneous epithelium were removed over the surgical area using tissue stainless-steel forceps (Dumont). A cranial window was made using micro dissecting spring (Roboz Surgical Instrument) and forceps in the skull of all fish, whether lesioned or sham lesioned. The cranial windows extended from the point of calibration (the midpoint of the intersection of the forebrain and the optic lobes) to the target lesion area. Since the rostral Dm is situated farther from the point of calibration compared to caudal Dm, the cranial window for rostral Dm lesions (~1 mm) was approximately twice the size of the window for caudal Dm lesions (~0.5 mm). This discrepancy in window size might have contributed to the lower rates of flaring observed in sham-rDm lesioned animals compared to sham-cDm lesioned animals.

Once the cranial window was formed, the DiI covered needle was zeroed at the calibration site. For cDm lesions, the needle was then shifted 200 μm anterior and 100 μm ventral to the zero point before aspiration. For rDm lesions, the needle was shifted 800 μm anterior and 100 μm ventral to the zero point. Since Dm is positioned in the medial most part of the forebrain, we performed bilateral lesions by aspirating at the midline with a needle of ~300 μm diameter. Aspirations were performed by gradually activating on the vacuum connected to aspiration needle while watching the tissue underneath a stereoscope to ensure proper removal. Following surgery, the small cranial window was sealed using a drop of kwik-sil (World Precision Instruments) and allowed to cure for 6–8 min. Fish were returned to their home tanks and allowed to recover for four days before post-surgery testing. Following post-surgery behavior, fish were euthanized and tissue was processed to determine the accuracy of the lesion. Four fish (16.7% of lesioned fish) were discarded due to lesions that were unilateral or impacted neighboring regions.

Telencephalic ablation surgeries were conducted using the same protocol as Dm lesions with the following exceptions. Prior to surgery, a pulled capillary needle was cut bluntly before being attached to a vacuum. The cranial window extended over the entirety of the telencephalon. Prior to aspiration of the telencephalon, a transverse incision was made across the entirety of the caudal extent of the telencephalon to sever any fiber bundles connecting the diencephalon. Inspection following aspiration ensured that optic nerves and optic lobes were not impacted by the surgery. Due to the size of the cranial window and invasiveness of the surgery, the survival rate for lesion and sham surgery was 37.5% and 50%, respectively.

##### Tracer injection

Fish were anesthetized using the method described above. To label retinal ganglion cells, 0.1% cholera toxin β (ctb-555; Invitrogen Cat#C34776 Lot# 2916405) was injected into the vitreous fluid of the eye through mouth pipetting (~10 nL), by inserting a capillary needle attached to an aspirator tube through a small hole made with spring scissors. Ctb was allowed to travel for 72 h before euthanasia and tissue processing.

To trace inputs to rDm and cDm, fish were anesthetized and intubated to flow tricaine over the gills during surgery, as described above. A cranial window was made extending from the point of calibration (the midpoint of the intersection of the forebrain and the optic lobes) to the target injection area. 0.1% ctb-555 was delivered unilaterally into either cDm (AP 200 μm from the point of calibration, ML 100 μm, DV 50–200 μm) or rDm (AP 800 μm, ML 100 μm, DV 50–200 μm). Injections were made via capillary needle connected to a Nanoject II (Drummond Scientific) injector at a flow rate of 1 nL/s with a total volume of 10 nL. Animals were allowed to recover for 48 h before euthanasia and tissue processing.

Fish were euthanized by submerging in ice water until immobile, followed by rapid decapitation. Heads were collected and fixed overnight in 4% paraformaldehyde in 0.1 M PB at 4°C. Brains were dissected out and further fixed in 4% paraformaldehyde overnight. Fixed brains were embedded in 3% low melting point agarose (Invitrogen, Cat# 16520–050) in PB and vibratome sectioned. Brain sections were stained with DAPI (5 μg/mL; Invitrogen, Cat#D1306) and imaged using a Yokogawa CSU-W1 spinning disk confocal microscope mounted on a Nikon Ti2-E stand with a 20 ×0.75 NA Plan Apochromat lambda objective (Nikon Instruments Inc). Brightness and contrast of images were adjusted using Fiji.^93^

### QUANTIFICATION AND STATISTICAL ANALYSIS

#### Quantifying aggressive display

We used a mix of manual and automated scoring of aggressive behaviors. Automated scoring was used to assess the proportion of time spent flaring in a large sample (*n* = 48 fish, 96 trials), which required a high-throughput method (Figures 1 and 2). However, when studying a smaller sample or when scrutinizing subtle changes in flaring scale, synchrony, or onset/offset timing, manual scoring was employed (Figures 3–6).

#### Manual scoring flaring bouts

Ground-truth flaring behavior was manually scored from the top-down video data using BORIS v. 7.9.24.^95^ Manual scoring was performed by a single individual blind to the behavior of the animation or the opponent. Flaring was divided into two sub-behaviors: partial flaring and full flaring. Partial flaring is present when the opercula are no longer touching the side of the body but not fully extended. Full flaring is when opercula reach and hold their maximum angle.

#### Unsupervised clustering

Unsupervised clustering of the geometrical features using tSNE followed by DBSCAN, as implemented in scikit-learn^96^ subdivided flaring into multiple subtypes dependent on features of the operculum, orientation, speed, turning angle, distance of head, centroid, and tail to stimulus, and tail angle and deviation (see Figure S1). However, these clusters are not completely discrete, as animals transition through intermediate geometries (Figure S16). Thus, for our analyses we focused on flaring state and its interaction with specific quantitative variables such as orientation.

#### Automated scoring

Two DeepLabCut (DLC)^38^ networks were trained to track body parts from the video data. The first network tracked 18 body parts from the top view (1,547 training frames from 20 individuals); the second network tracked 15 points from the side view (802 training frames from 15 individuals; Figure S1B). Predicted markers with confidence values less than a threshold of 0.8 or a “jump” from a previous frame larger than 15 pixels were dropped, and linear interpolation was used to fill the missing data. Separately, the contours of the fish were computed from the raw video data using a custom OpenCV script (Figure S1C; Data S2). To fit a contour to fish, first the background was subtracted from each frame to create a binary image. The largest contour was found in the binarized image. The point in the contour closest to the DLC head and tail markers were considered the head and tail, respectively, of the contour. Lines were fit to the 100 points surrounding the marked head and tail to determine the tail’s angle and how far it deviates from the body line. Behaviorally-relevant features were then extracted from the DLC markers (Figure S1D) and the contour (Figure S1E), including: orientation, speed, turning angle, head (x, y, z), centroid (x, y, z), tail (x, y), tail angle, tail deviation, and features of the operculum (operculum angle, left and right operculum angles and distance from midline). All features were smoothed using a median filter with a window of 11 frames (0.275 s). These behavioral features, and their associated labels from manual scoring, were then used to train a model that classifies the features into one of three available behavior classes for each time point: (1) no flaring; (2) partial flaring; (3) full flaring. The model^39^ is a dilated Temporal Convolutional Network (dTCN),^97^ which has shown good performance on similar behavioral classification tasks^39,98,99^ (Figure S1F).

#### dTCN architecture and loss

The dTCN model is composed of multiple “Dilation blocks.” Each Dilation block is composed of two 1D convolution layers (filter size of 9-time steps and 32 channels per layer), each of which is followed by a leaky ReLU activation function and weight dropout with probability *p* = 0.1. An additional residual path bypasses the convolution layers and is combined with their output before the application of a final leaky ReLU function. The input features are passed through two such Dilation blocks, and the output of the final Dilation block is passed through a final dense layer followed by a softmax function to produce class probabilities. The model is trained using a standard cross-entropy loss function between the predicted class probabilities and the ground truth scores.^100^ Because of the class imbalance present in this dataset, less frequent classes have higher weights in the loss function: if the total number of labeled frames is N, and the number of labeled frames for class i is $N_{i}$, the loss for data points from class is weighted by $N/N_{i}$.

#### dTCN training

Model training utilized 21 videos of individuals which contained a total of 1,512,000 hand scored frames (no flaring: 1,175,298 frames; half flaring: 177,978 frames; full flaring: 158,724 frames). These training videos were a mix of encounters with animations and conspecifics. The data were split into temporal sequences of 1,000 time points, and each training batch contained 8 such sequences. 90% of sequences were used for training, 10% for validation. Training terminated once the loss on validation data began to increase for 20 consecutive epochs; the epoch with the lowest validation loss was used for evaluation. All models were trained with the Adam optimizer using an initial learning rate of 10^−4^.

#### dTCN evaluation

Model evaluation utilized 19 videos of held-out individuals which contained a total of 1,368,000 hand scored frames. The argmax of the predicted class probabilities at each time point was used to construct ethograms of behavioral state (Figure S1G,H). To evaluate the models, the F1 score —the geometric mean of precision and accuracy— was computed for all behaviors across the 19 held-out test individuals (Figure S1I). Held-out test individuals were comprised solely of virtual fish encounters and therefore had overall higher flaring rates, compared to training set. Because F1 score is correlated with proportion time flaring, this led to a higher average F1 score for withheld trials compared to training trials (Figure S1J).

A coarser measure of model performance related to the analysis in Figure S1 compares the proportion of time spent flaring (combining half and full flaring) predicted by the model and by hand scoring, for both training trials and held-out test trials (Figures S1K and S1L). All figures and supplemental figures visualize “combined” flaring, in which half and full flaring count as flaring.

#### Persistence of flaring and other metrics

To quantify the proportion time flaring within a given sub-epoch within exposure period, the persistence of flaring for each trial was calculated by first binning the flaring time series into 8-s bins. A rolling mean window (width 0.357 s) was applied to the bins. The average persistence across individuals was calculated and an exponential curve was fit to the average proportion time flaring. The timepoint in which the fit exponential curve becomes ¾ (Figures 2 and 4) or ½ (Figure 6) its maximum height is calculated. Similarly, for position and orientation, we calculated averages using 8-s bins with the same rolling mean window used for flaring persistence.

#### Synchronization toward animations

To measure synchronization, the flaring frequency $\left(X_{S}\right)$ at time t in the animation was calculated by adding flaring responses to each loop of the animation $\left(x_{i}\right)$ at time t and dividing by the number of loops of the animation (S) (see Equation 1).

The variance $\left(\sigma_{X_{S}}^{2}\right)$ of the distribution of frequencies was then calculated (see Equation 2). If a fish displays high flaring frequencies toward the animation at certain times and low flaring frequencies at other times, instead of a uniform flaring frequency throughout, they are considered synchronized and will have a larger variance of frequencies.


(Equation 1) $$X_{S}(t)=\frac{\sum_{i=0}^{s}\;\;x_{i}(t)}{S}$$

(Equation 2) $$\sigma_{X_{S}}^{2}=\frac{\sum {\left(X_{S}(t)\;-\;\overset{-}{X_{S}}\right)}^{2}}{T\;-\;1}$$

To determine whether fish are significantly synchronized to the animation, compared to chance, we performed a resampling of randomly offset controls (see Equation 3). Flaring responses to each loop of the animation *(*$x_{i}$) was shifted by a random value (θ) within the range of 0 to the length of the animation (T). With each flaring response randomly offset, we calculated the new variance of the frequencies $\left(\sigma_{X_{s}^{\prime }}^{2}\right)$.

Sample $\sigma^{2}\;\sim \;P_{\mathrm{null}}\left(\sigma^{2}\right)$ (see below).

***For*** i=0…S

***Sample*** θ∼Unif(0,T)

$x_{i}^{\prime }=shift\left(x_{i},\theta \right)$

$$X_{S}^{\prime }(t)=\frac{\sum_{i=0}^{S}\;x_{i}^{\prime }(t)}{S}$$

(Equation 3) $$\sigma_{X_{s}^{\prime }}^{2}=\frac{\sum \;{\left(X_{s}^{\prime }(t)\;-\;\overset{-}{X_{s}^{\prime }}\right)}^{2}}{T\;-\;1}$$

We repeated this resampling for 1,000 iterations to create a set of variances that can occur by chance while still maintaining the structure of the behavior. We calculated the 95^th^ percentile of the distribution of variances $\left(\sigma_{thresh}^{2}\right)$. If the actual variance ($\sigma_{X_{S}}^{2}$) was greater than $\sigma_{\mathrm{thresh}}^{2}$, we concluded that the fish was significantly synchronized to the animation.

Calculate $\sigma_{\mathrm{thresh}}^{2}\;s.t$.


(Equation 4) $$P_{\mathrm{null}}\left[\sigma_{x_{s}}^{2}>\sigma_{\mathrm{thresh}}^{2}\right]=0.05$$

If $\sigma_{X_{s}}^{2}>\sigma_{\mathrm{thresh}}^{2}$, reject the null hypothesis.

##### Correlations of flaring and stimulus behavior

Features of shape and motion were gathered from both opponents during conspecific encounters or for one opponent during animation encounters. Subsequently, we filtered time series data to include periods when either opponent was flaring during conspecific interactions, or when the opponent or animation was flaring during animation encounters. This step aimed to prevent artificially inflating high correlations of flaring observed in low-flaring dyads.

Next, we conducted point biserial correlations between flaring and opponent or animation features. In cases in which fish did not flare, we assigned a correlation value of 0 with all features. As a control, we rearranged the filtered time series of features in a random order and recalculated the correlation between flaring and each feature. A random shuffle, as opposed to a circular shift (as was done in quantifying synchronization toward animation above), was used since the correlation metric does not depend on temporal structure. We conducted paired *t*-tests between each population of actual versus shuffled correlation coefficients to assess the significance of the correlation.

##### Peri-event time histograms

We aligned the behavior of either the opponent fish or the animated stimulus with the onset or offset of a flaring bout exhibited by the focal opponent. Specifically, we chose flare bout onsets where a fish had not flared within the preceding 3 s and continued to flare for the subsequent 3 s. Similarly, for flare bout offsets, we selected instances where a fish had been flaring for 3 s prior and did not flare again for 3 s afterward. This selection criterion aimed to filter out flaring bouts that were either too brief or occurred in too close succession, minimizing potential noise in the opponent’s response.

To calculate the elevation peri-event time histogram across time periods surrounding multiple bout onsets and offsets, we first conducted a normalization step on a period-by-period basis (Figures 3F and 3G). The elevation in the period surrounding each bout onset or offset was normalized by subtracting the minimum elevation within the period (3 s before and three after the onset or offset) and then dividing by the adjusted maximum elevation within that period. This normalization step accounted for absolute variations in elevation across different periods.

##### Creating directional transition matrices

Transition networks were created by segmenting the behavior of both the fish and the animation into eight discrete states, defined by their flaring and orientation status (facing, turning, lateral, and turning away). The state durations were exponentially distributed (Figure S17). Combinatorial states were then formed between each fish and their opponent (real or virtual), resulting in a total of 64 possible states. Data from all dyads were combined to generate a population-level directional transition matrix for real fish encounters, and the same was done for animation encounters.

We calculated the transition probabilities between each of these 64 states. Due to the high number of states and transitions, we applied a filtering step to simplify the visualization. First, we filtered out any state where fish spent less than 2% of their time. This reduced the number of states to 24 for real fish encounters and 18 for animation encounters. Next, we removed transitions with a transition probability of less than 0.0025 in both conditions.

We then created a directional transition matrix, clustered using a spectral layout,^101^ which grouped states based on the first and second eigenvectors of the transition matrix and strength of their transition probabilities. The circular nodes in the visualization represent each state, with node size corresponding to the proportion of total time the fish spent in that state. The thickness of the arrow lines between nodes represents the transition probabilities between states.

## Supplementary Material

1

2

4

5

6

7

8

9

10

11

## Figures and Tables

**KEY RESOURCES TABLE:** 

REAGENT or RESOURCE	SOURCE	IDENTIFIER

Antibodies		

Rabbit anti-pS6 (1:10,000)	ThermoFisher	Cat# 44-923G, Lot# 2066361; RRID: AB_2533798

Chemicals, peptides, and recombinant proteins		

NeuroTrace	ThermoFisher	Cat# N21480; RRID: AB_2620170
ctb (cholera toxin β subunit) 555	Invitrogen	Cat# C34776, Lot# 2916405
Dil	Invitrogen	Cat# D3911
Kwik-sil	World Precision Instruments	N/A

Deposited data		

Behavior videos	Dryad	https://doi.org/10.5061/dryad.7wm37pw2w
Manual scoring of behavior videos	Dryad	https://doi.org/10.5061/dryad.7wm37pw2w
Extracted features of behavior videos	Dryad	https://doi.org/10.5061/dryad.7wm37pw2w
Custom animations	Dryad	https://doi.org/10.5061/dryad.7wm37pw2w

Software and algorithms		

Original code	Dryad	https://doi.org/10.5061/dryad.7wm37pw2w

Other		

Blender v2.91	https://www.blender.org/	Blender – A 3D modeling and rendering package. Stichting Blender Foundation, Amsterdam.
BORIS v. 7.9.24	https://github.com/olivierfriard/BORIS	https://doi.org/10.1111/2041-210X.12584
DeepLabCut v2.3	https://github.com/deeplabcut	https://doi.org/10.1038/s41593-018-0209-y
Daart	https://github.com/themattinthehatt/daart	https://doi.org/10.1101/2021.06.16.448685
FIJI2 v 2.1.0/1.53c	https://imagej.net/software/fiji/	https://doi.org/10.1038/nmeth.2019
Tools for behavioral analysis	This study	https://doi.org/10.5061/dryad.7wm37pw2w

## Highlights

- Betta take turns in ritualistic visual agonistic interactions before engaging in fight
- Specific visual cues coordinate turn taking, while others prolong the agonistic interaction
- Agonistic turn taking does not require the telencephalon
- The pallial amygdala prolongs aggressive states
