# Supplementary material for: Coordination and persistence of aggressive visual communication in Siamese fighting fish
Source: Cell Rep. Author manuscript; Available in PMC 2025 Feb 19. (PMC11837226; doi:10.1016/j.celrep.2024.115208)
Supplement: 1 [file NIHMS2052777-supplement-1.pdf]

Cell Reports, Volume *44*

Supplemental information

Coordination and persistence of aggressive visual communication in Siamese fighting fish

Claire P. Everett, Amy L. Norovich, Jessica E. Burke, Matthew R. Whiteway, Paula R. Villamayor, Pei-Yin Shih, Yuyang Zhu, Liam Paninski, and Andres Bendesky

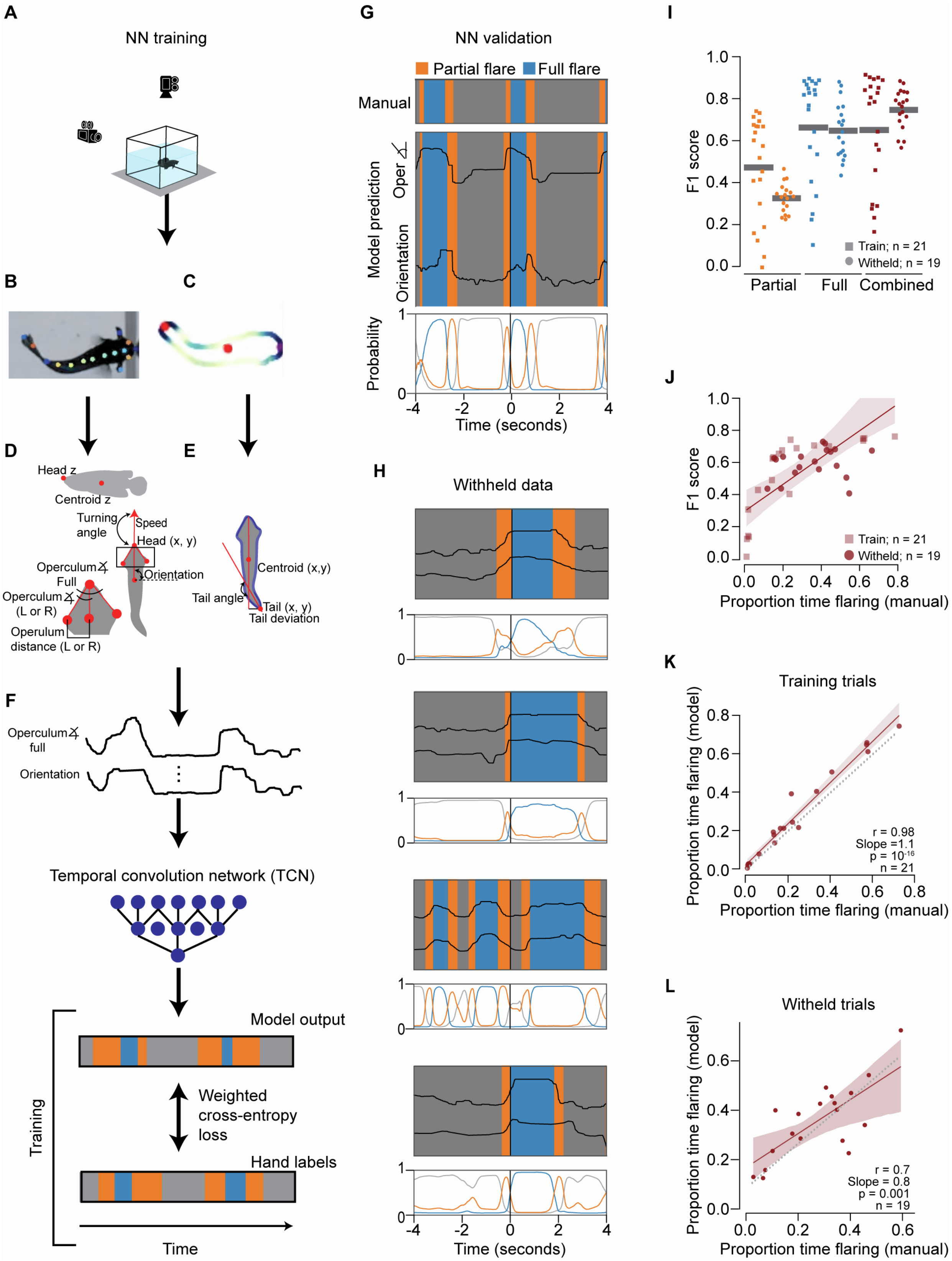
A
NN training
B
C
D
Head z
Centroid z
Turning angle
Speed
Head (x, y)
Operculum ∡ Full
Orientation
Operculum ∡ (L or R)
Operculum distance (L or R)
E
Centroid (x,y)
Tail angle
Tail (x, y)
Tail deviation
F
Operculum ∡ full
Orientation
Temporal convolution network (TCN)
Model output
Weighted cross-entropy loss
Hand labels
Training
Time
G
NN validation
Partial flare
Full flare
Manual
Model prediction
Oper ∡
Orientation
Probability
Time (seconds)
H
Withheld data
Time (seconds)
I
F1 score
Train; n = 21
Witheld; n = 19
Partial
Full
Combined
J
F1 score
Train; n = 21
Witheld; n = 19
Proportion time flaring (manual)
K
Training trials
Proportion time flaring (model)
r = 0.98
Slope =1.1
p = 10^-16
n = 21
Proportion time flaring (manual)
L
Witheld trials
Proportion time flaring (model)
r = 0.7
Slope = 0.8
p = 0.001
n = 19
Proportion time flaring (manual)

Figure S1: *daart* pipeline for automated scoring of flaring bouts. (A) Video setup of betta recorded from the top and side. **(B)** Fish body parts tracked using markerless pose estimation. **(C)** Contour of the body tracked. **(D, E)** Features of shape and motion extracted using **(D)** tracked body parts or **(E)** contour. **(F)** Temporal convolution network (TCN) model training pipeline. **(G)** Comparison of manually-scored flaring events (top), model-scored flaring events (middle), and probability of fish being in each state (background, full, partial flare) according to TCN (bottom). **(H)** Examples of model-scored flaring events in withheld trials. **(I)** F1 scores of training and withheld trials for three states (partial flaring, full flaring, and combined partial and full flaring). **(J)** Correlation of proportion time flaring scored manually with F1 score. **(K, L)** Correlation of proportion time flaring scored manually with times by model for training trials **(K)** and withheld trials **(L)**. **(I-L)** each dot denotes a trial.

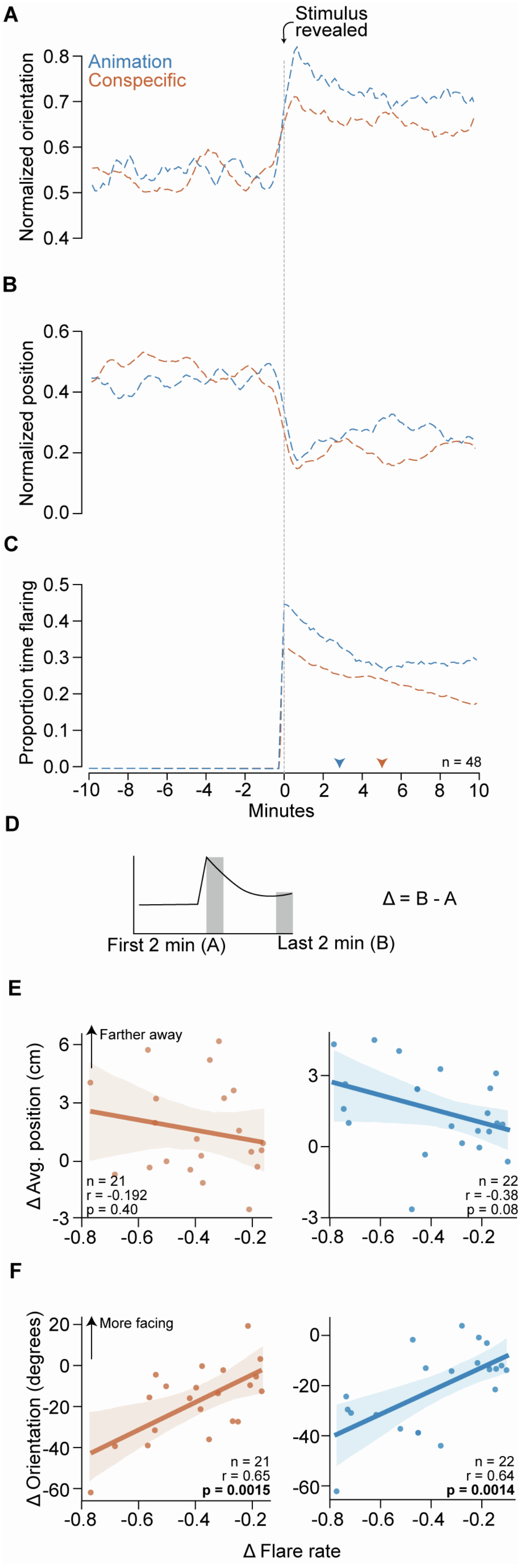

Figure S2: Changes of position and orientation through trials. **(A-C)** Changes in average orientation **(A)**, position **(B)**, and flare rate **(C)** across habituation and exposure period. **(D)** Flare rate, and average position and orientation were taken for the first and last 2-minute windows of the exposure period (grey boxes) and subtracted to compute the change (Δ) over the exposure period. **(E, F)** Correlations of the change in flare rate with position (**E**) and orientation (**F**) in conspecific (left) and animation (right) encounters.

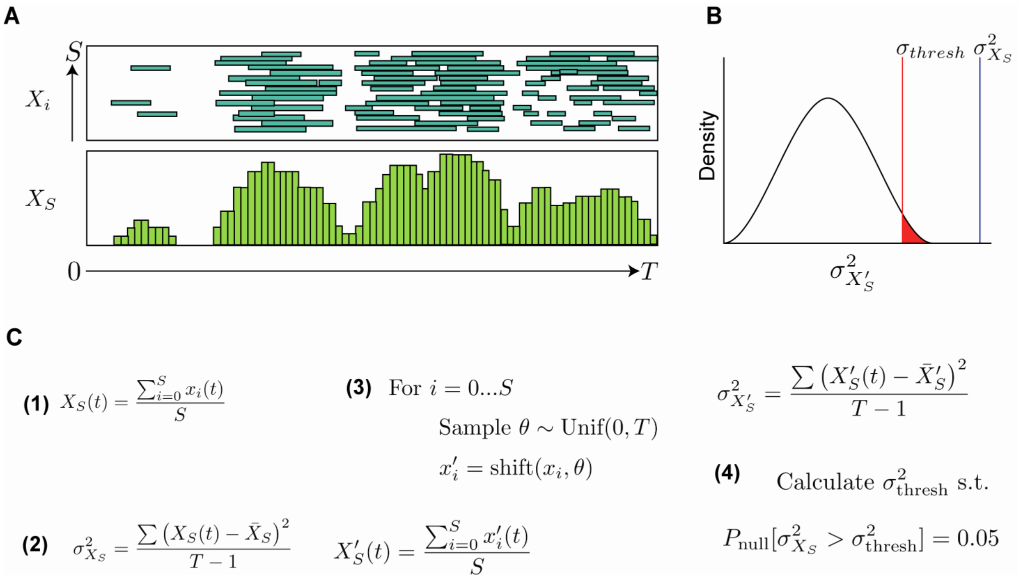

Figure S3: Quantifying synchronization with animations. (A) Flare responses (X_i) in each loop (S) of the animation (top) and the flaring frequency (X_s) towards each timepoint (t) in the animation (bottom). **(B)** Distribution of variance of flaring frequency by chance ($\sigma^2_{X'_S}$) with a sample actual variance of flaring frequency ($\sigma^2_{X_S}$) falling significantly outside that distribution, denoting synchronization. **(C)** Equations calculating: the flaring frequency at time t (1), the variance of flaring frequencies (2), a set of variances of flaring frequency due to chance (3), whether the actual variance of flaring frequencies falls significantly outside of variance due to chance (4).

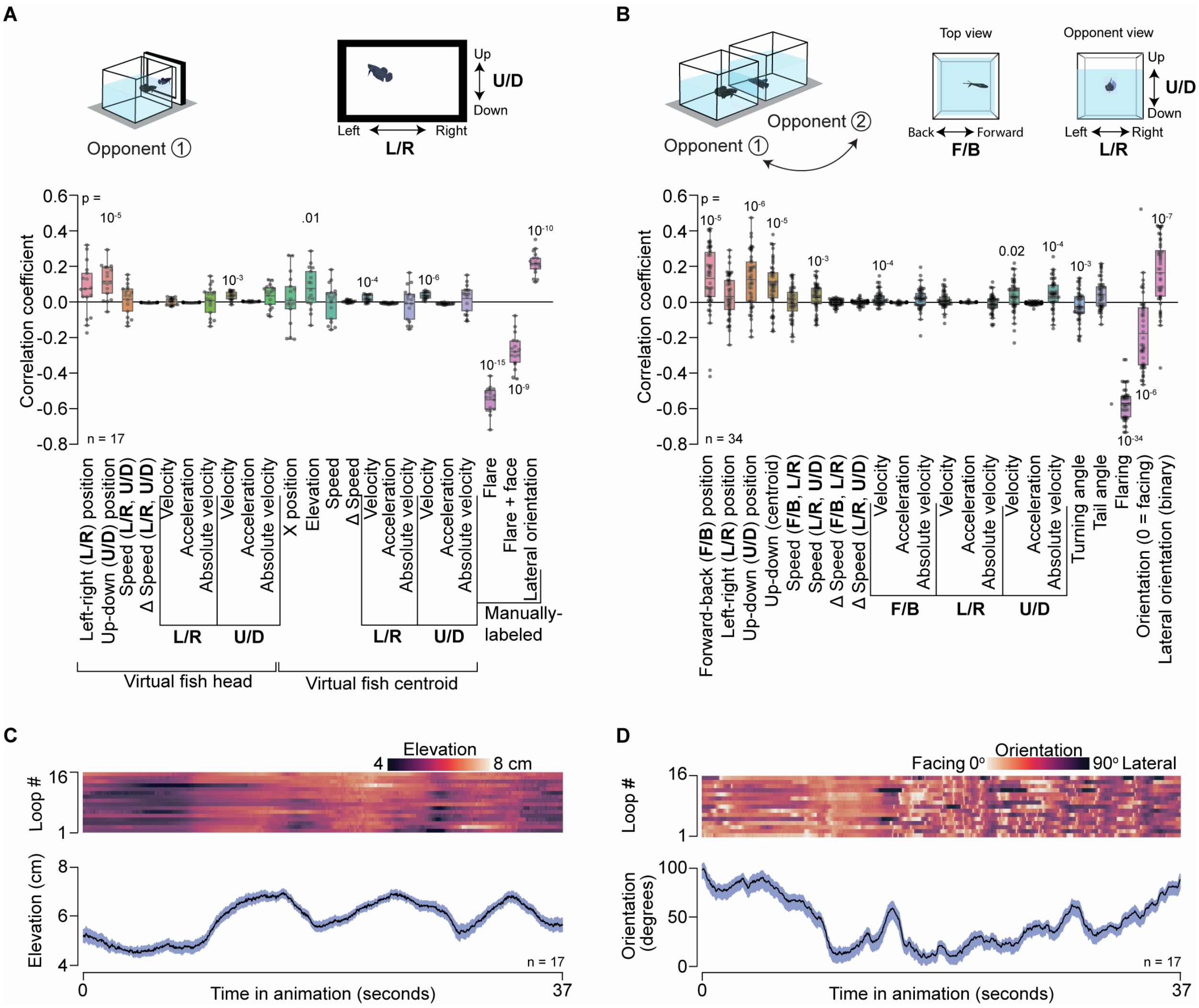

Figure S4: Betta coordinate their flare response, orientation, and elevation to multiple dynamic visual cues emitted by naturalistic animations and conspecifics. (A, B) Correlation between flaring of real fish with that of the animation **(A)** or their opponent **(B)**. p-values by paired t-test comparing correlations to shuffled controls. **(C)** Elevation and **(D)** orientation of an individual exposed to animation (top) and population average ±SEM (bottom). These are additional analyses of the behavior tests presented in Fig. 3.

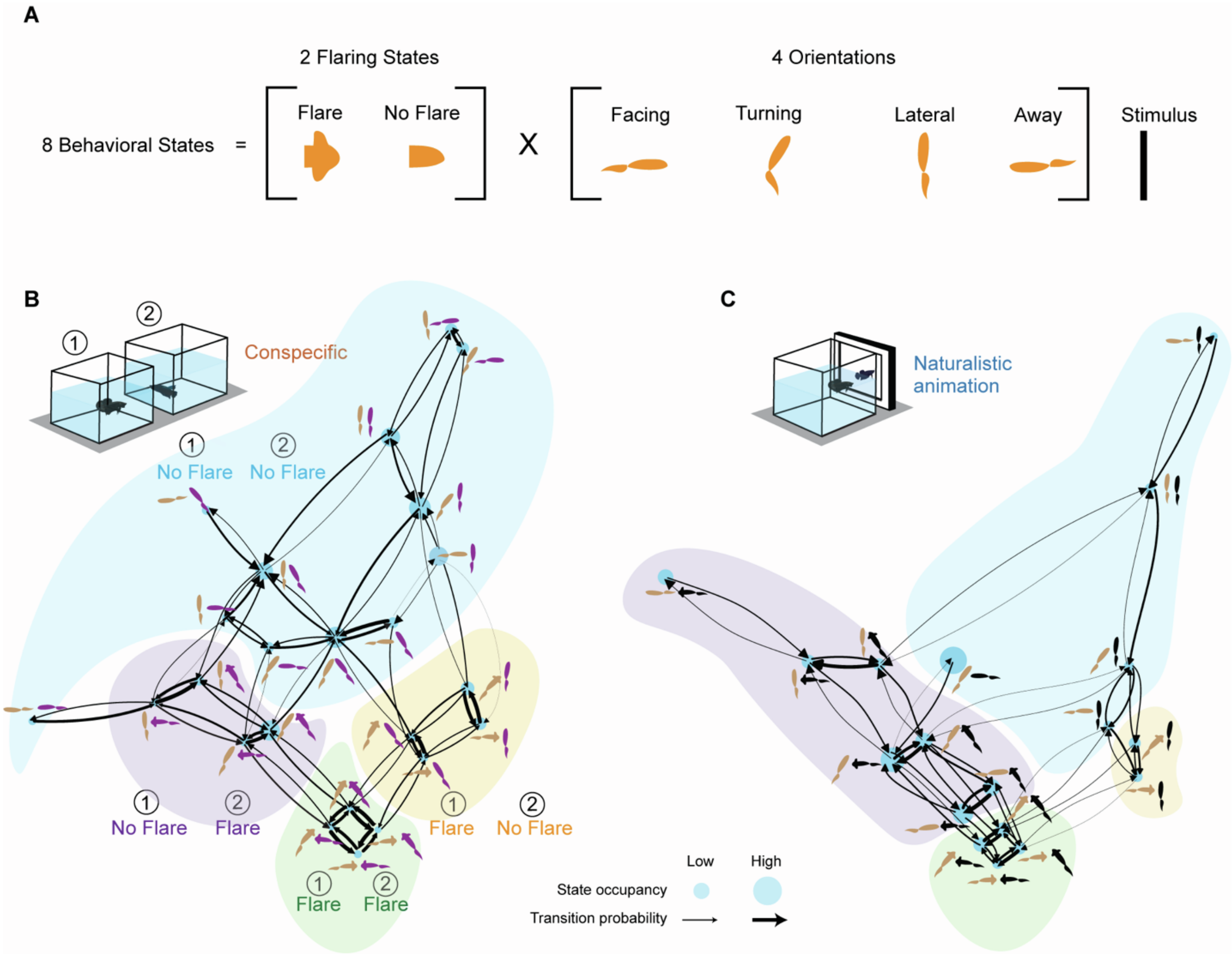

Figure S5: Behavior transition networks during conspecific or animation encounters. (A) Behavioral states segmented by flaring state and orientation. Each fish can be in 8 states, which in combination with the state of the conspecific or animated fish yield 64 states. **(B,C)** Transition networks for conspecific **(B)** and animation **(C)** encounters including states that occur at least 2% of the time and transitions with a probability greater than 0.0025. Both networks adopted similar geometric configurations and shared many behavioral sequences. Transition probabilities were widely distributed without a particular pathway leading to dominant flaring behavior against either conspecific or animation. The "purple" group of states in the animation encounters was more prominent than in conspecific encounters, consistent with an animation whose behavior doesn't respond to the real fish. Certain trends emerge in both networks; for example, when only one fish is flaring (as seen in the yellow or purple groups), the non-flaring fish was most likely to be lateral or turning, never directly facing the other. This observation aligns with the patterns of turn-taking in betta displays we describe in other figures.

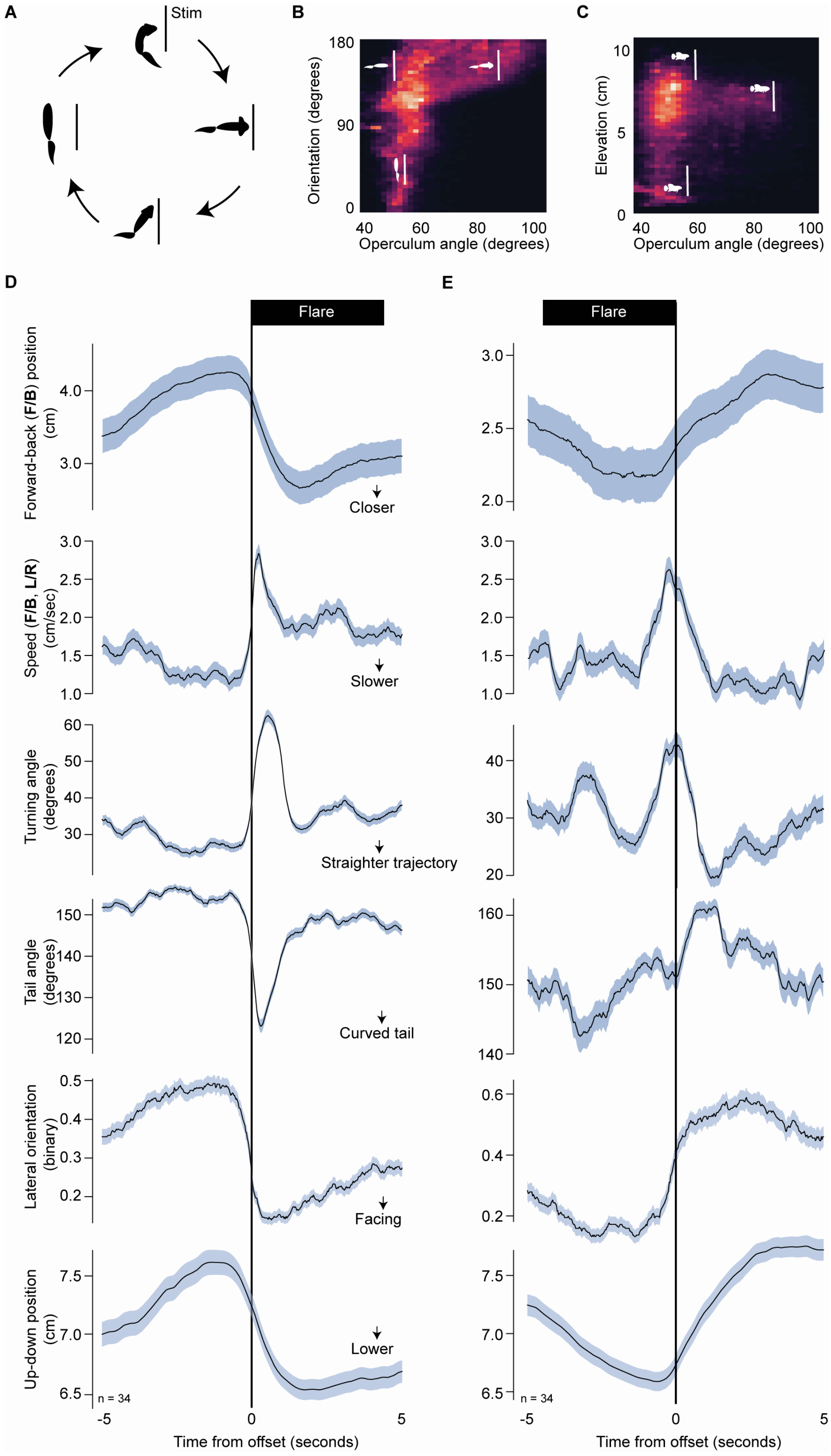

A
Stim
B
180
90
0
Orientation (degrees)
40
60
80
100
Operculum angle (degrees)
C
10
5
0
Elevation (cm)
Operculum angle (degrees)
D
Flare
Forward-back (F/B) position (cm)
Closer
Speed (F/B, L/R) (cm/sec)
Slower
Turning angle (degrees)
Straighter trajectory
Tail angle (degrees)
Curved tail
Lateral orientation (binary)
Facing
Up-down position (cm)
Lower
n = 34
Time from offset (seconds)
E
Flare
n = 34
Time from offset (seconds)

Figure S6: Multiple behavioral changes co-occur as betta begin or terminate flaring. (A) Betta transition between “phases”. Phase starting at the left and moving clockwise: (1) betta lateral and not flaring, (2) betta beginning to extend gills and turn to face, (3) betta flaring and facing, (4) betta beginning to retract gills and turning laterally. **(B, C)** 2D histograms of fish’s operculum angle and orientation **(B)** or elevation **(C)** show that fish mostly flare while facing and are more elevated. White overlayed diagrams schematize betta posture and position. **(D, E)** Peri-event time histograms (mean±SEM) of within-fish dynamics at the onset (left) or offset (right) of flaring bouts.

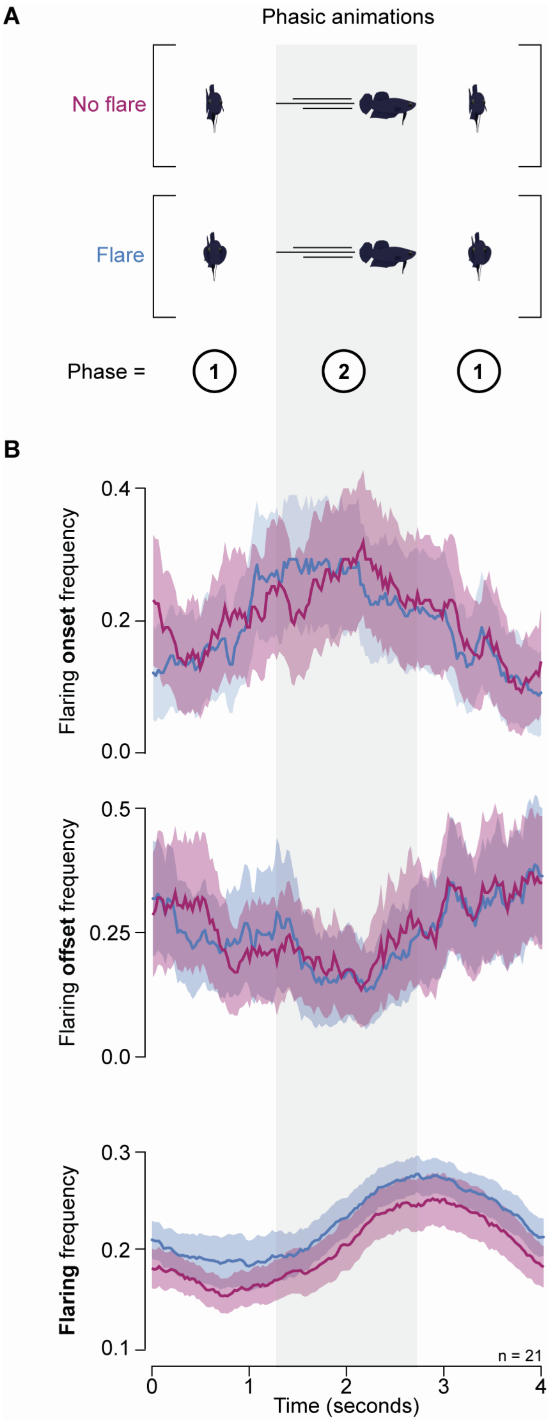

Figure S7: Betta coordinate their flaring with biphasic animations. (A) Animations were composed of two alternating phases with distinct combination of flare, orientation, and speed state. **(B)** Frequency of flaring bout onsets (top), offsets (middle), and overall flaring frequency (bottom) aligned to a loop of the animation.

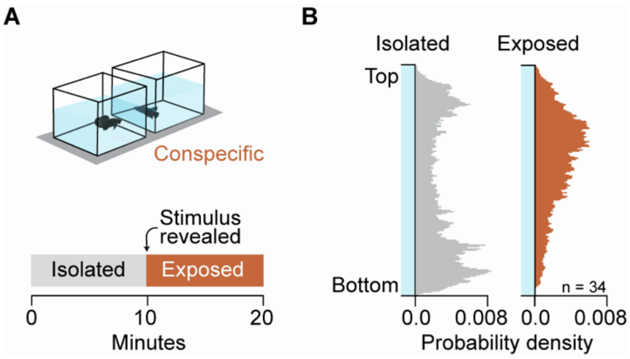

Figure S8: Betta elevate themselves in the water column when exposed to another individual. (A) Conspecific paradigm: two opponents in neighboring tanks. **(B)** Elevation distribution during isolation (left) and exposure (right) to opponent.

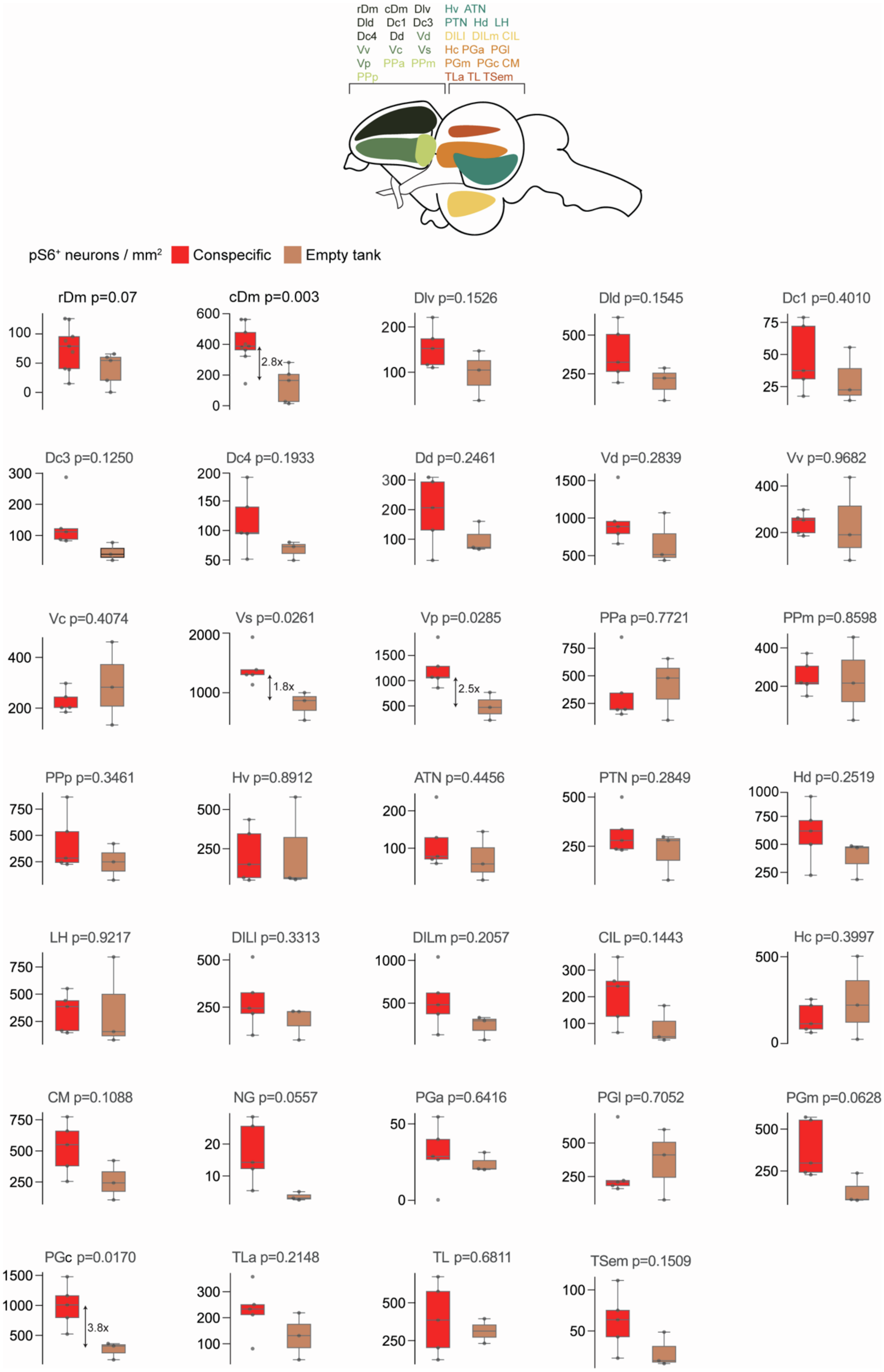
rDm cDm Dlv
Dld Dc1 Dc3
Dc4 Dd Vd
Vv Vc Vs
Vp PPa PPm
PPp
Hv ATN
PTN Hd LH
DILl DILm CIL
Hc PGa PGl
PGm PGc CM
TLa TL TSem
pS6⁺ neurons / mm²
Conspecific
Empty tank
rDm p=0.07
cDm p=0.003
2.8x
Dlv p=0.1526
Dld p=0.1545
Dc1 p=0.4010
Dc3 p=0.1250
Dc4 p=0.1933
Dd p=0.2461
Vd p=0.2839
Vv p=0.9682
Vc p=0.4074
Vs p=0.0261
1.8x
Vp p=0.0285
2.5x
PPa p=0.7721
PPm p=0.8598
PPp p=0.3461
Hv p=0.8912
ATN p=0.4456
PTN p=0.2849
Hd p=0.2519
LH p=0.9217
DILl p=0.3313
DILm p=0.2057
CIL p=0.1443
Hc p=0.3997
CM p=0.1088
NG p=0.0557
PGa p=0.6416
PGl p=0.7052
PGm p=0.0628
PGc p=0.0170
3.8x
TLa p=0.2148
TL p=0.6811
TSem p=0.1509

Figure S9: pS6 activity map of areas differentially activated during aggressive display. rDm: rostral portion of the dorsal medial area of the pallium, cDm: caudal portion of the dorsal medial area of the pallium, Dlv: ventral division of the lateral zone of the dorsal telencephalon, Dld: dorsal division of the lateral zone of the dorsal telencephalon, Dc: central zone of the dorsal telencephalon, Dd: dorsal zone of the dorsal telencephalon, Vd: dorsal nucleus of the ventral telencephalon, Vv: ventral nucleus of the ventral telencephalon, Vc: central nucleus of the ventral telencephalon, Vs: supracommissural nucleus of the ventral telencephalon, Vp: postcommissural nucleus of the ventral telencephalon, PPa: anterior parvocellular preoptic nucleus, PPm: magnocellular preoptic nucleus, PPp: posterior parvocellular preoptic nucleus, Hv: ventral zone of periventricular hypothalamus, ATN: anterior tuberal nucleus, PTN: posterior tuberal nucleus, Hd: dorsal zone of periventricular hypothalamus, LH: lateral hypothalamic nucleus, DILl: lateral diffuse nucleus of the inferior lobe, DILm: medial diffuse nucleus of the inferior lobe, CIL: central diffuse nucleus of the inferior lobe, Hc: caudal zone of periventricular hypothalamus, CM: corpus mammilare (mamillary body), PGa: anterior preglomerular nucleus, PGl: lateral preglomerular nucleus, PGm: medial preglomerular nucleus, PGc: commissural preglomerular nucleus, TLa: anterior torus semicircularis, TL: torus longitudinalis, TSem: torus semicircularis. p-values by unpaired t-test. Nomenclature informed by betta[1,2], cichlid[3], and zebrafish[4] atlases.

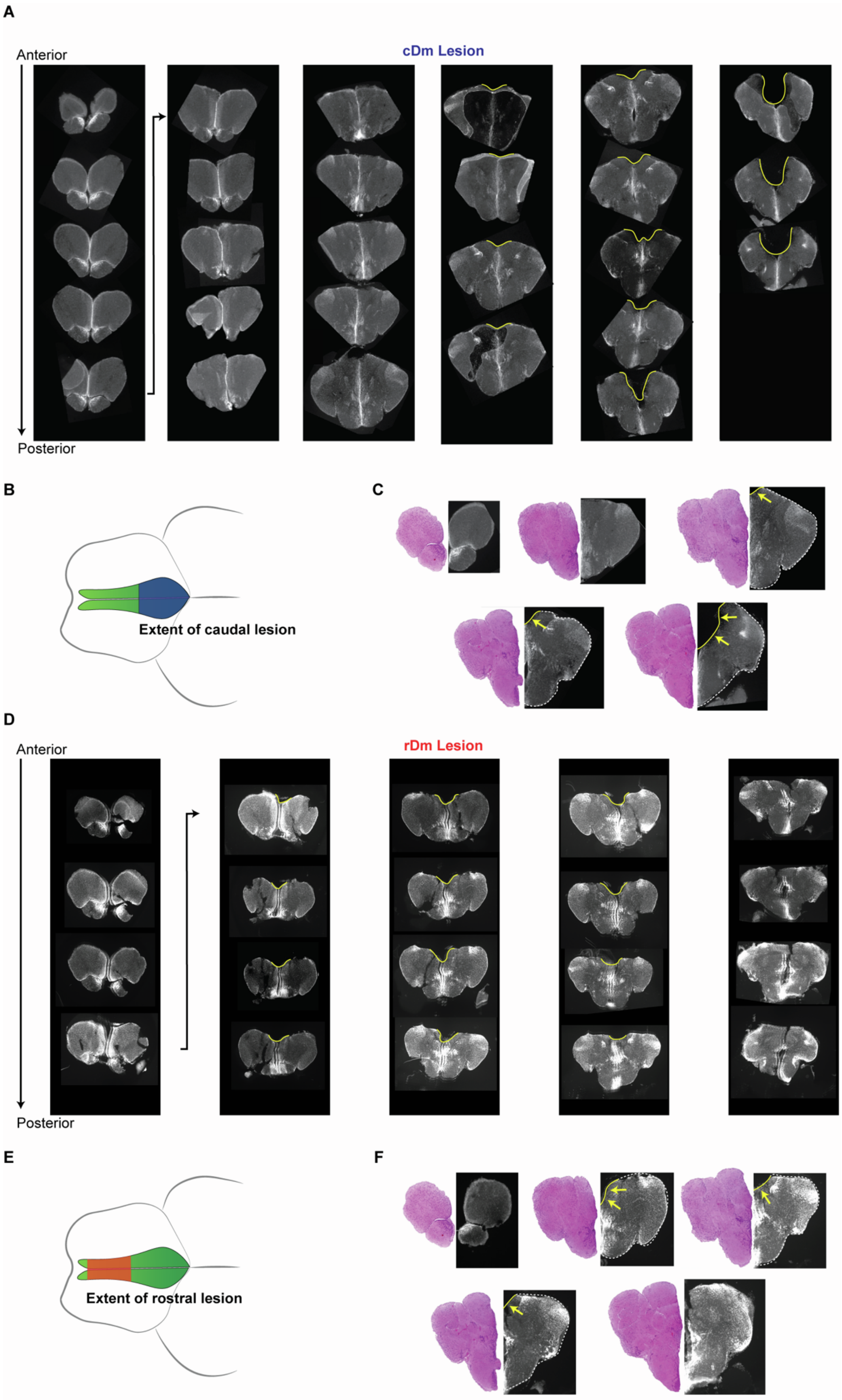
A
Anterior
cDm Lesion
Posterior
B
Extent of caudal lesion
C
D
Anterior
rDm Lesion
Posterior
E
Extent of rostral lesion
F

Figure S10: Histology of Dm lesions. (A,D) Coronal sections of forebrain with cDm **(A)** or rDm **(D)** lesions. **(B,E)** Diagrams of extent of lesions along anteroposterior axis. **(C,F)** Select sections from series aligned to betta brain atlas[1]. Arrows point to the brain region removed by the lesion.

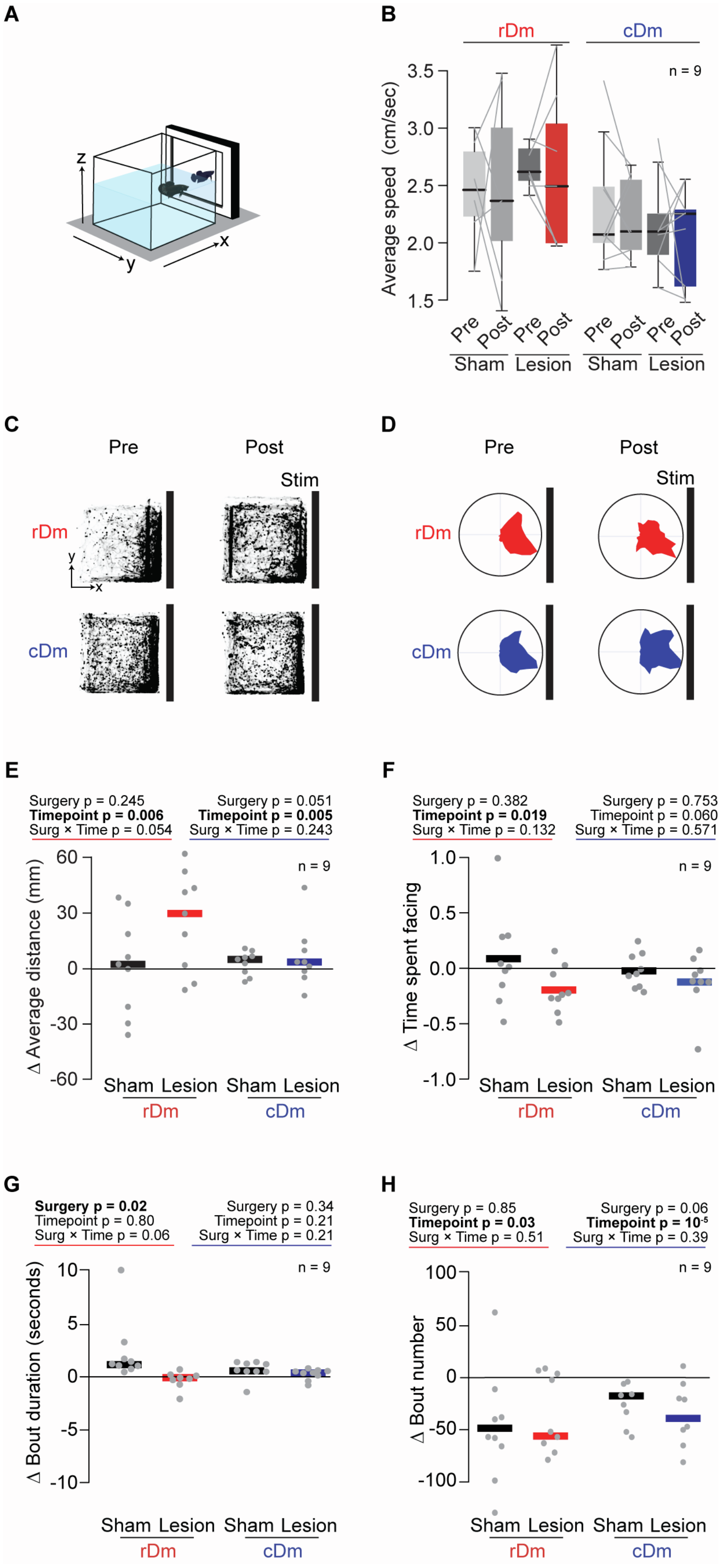

Figure S11: Additional metrics of behavior towards animation following lesions. (A) Naturalistic animation paradigm: one opponent faces a naturalistic animation modeled after a male displaying aggression. **(B)** Average speed during free swimming before the animation starts pre- and post- rDm (left) and cDm (right) lesions and associated sham controls. **(C)** Heatmaps showing tank occupancy (from a top view) by fish during exposure pre- and post-lesion. **(D)** Polar plots showing distributions of the head orientation during exposure pre- and post-lesion. **(E)** Change (post-pre surgery) in the distance to the stimulus. **(F)** Change (post-pre surgery) in the proportion time spent facing within 180º of the stimulus. **(G)** Change (post-pre surgery) in flare bout duration. **(H)** Change (post-pre surgery) in the number of flare bouts. **(E-G)** Dots denote individuals with lines at the median. p-values by mixed models ANOVA.

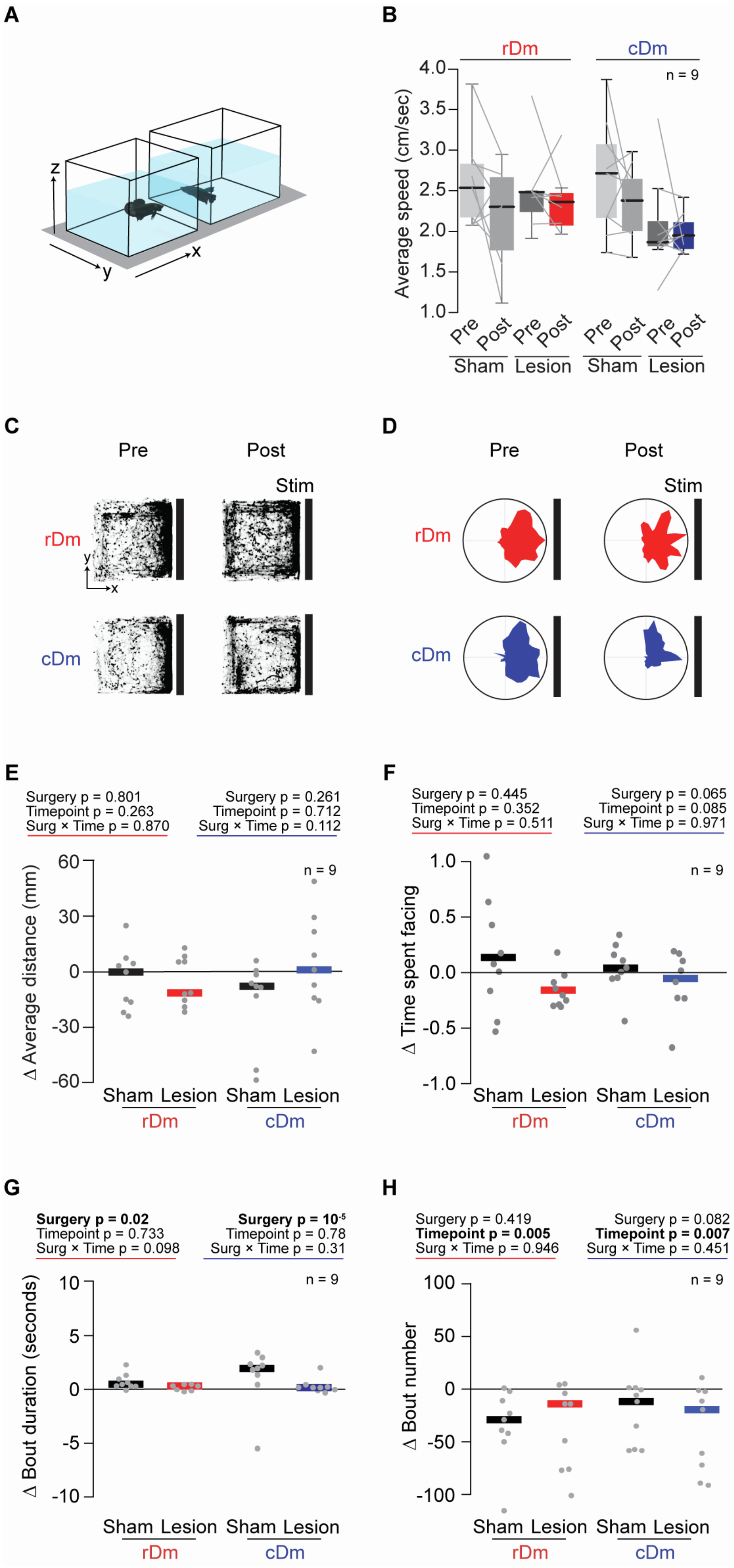

Figure S12: Additional metrics of behavior towards conspecific following lesion. (A) Conspecific paradigm: two opponents in neighboring tanks. **(B)** Average speed during free swimming before exposure to conspecific pre- and post- rDm (left) and cDm (right) lesions and associated sham controls. **(C)** Heatmaps showing tank occupancy (from a top view) by fish during exposure pre- and post-lesion. **(D)** Polar plots showing distributions of the head orientation during exposure pre- and post-lesion. **(E)** Change (post-pre surgery) in the distance to the stimulus. **(F)** Change (post-pre surgery) in the proportion time spent facing within 180º of the stimulus. **(G)** Change (post-pre surgery) in flare bout duration. **(H)** Change (post-pre surgery) in the number of flare bouts. **(E-G)** Dots denote individuals with lines at the median. p-values by mixed models ANOVA.

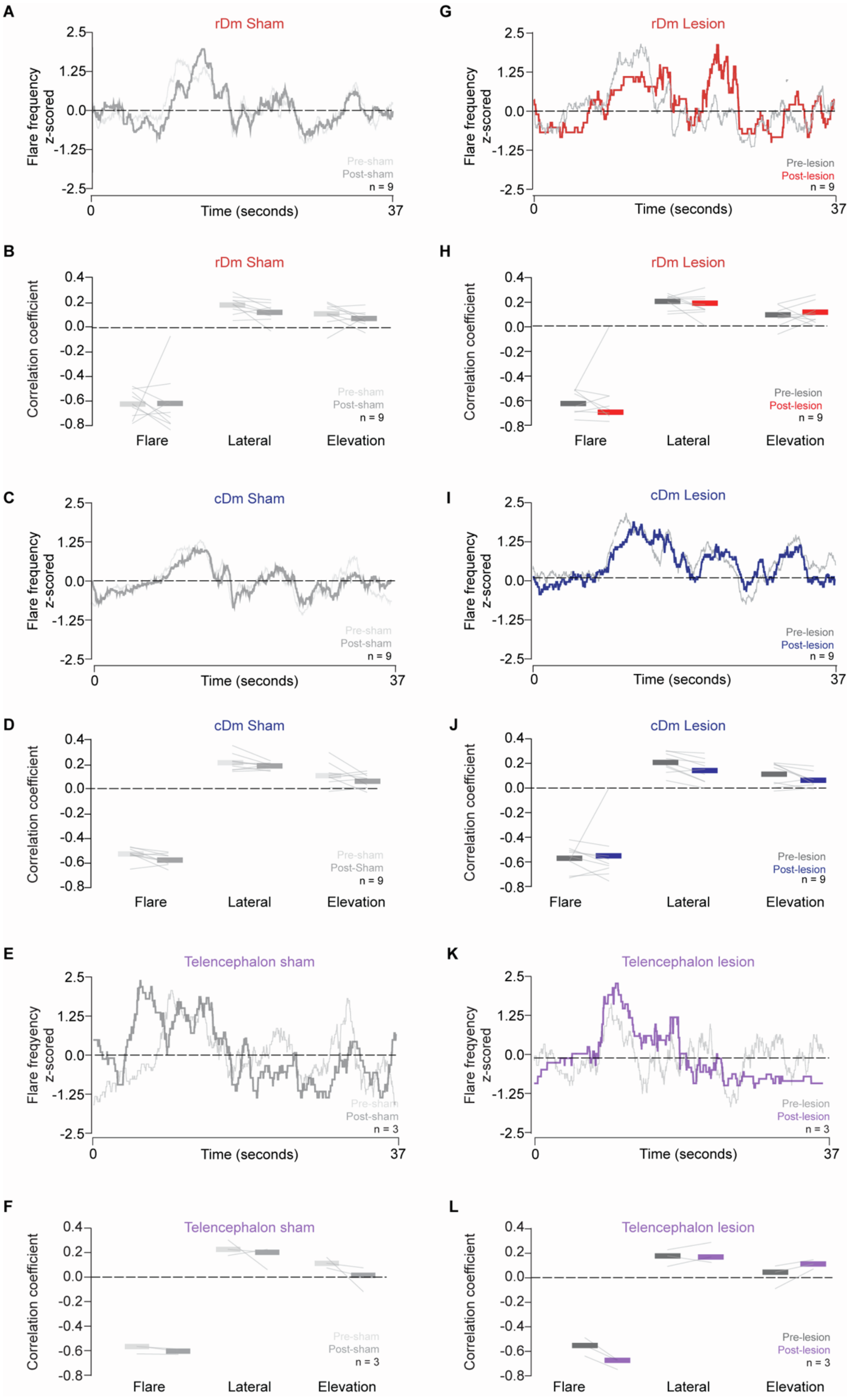

A
rDm Sham
Flare frequency z-scored
Time (seconds)
Pre-sham
Post-sham
n = 9
B
rDm Sham
Correlation coefficient
Flare
Lateral
Elevation
C
cDm Sham
D
cDm Sham
Post-Sham
E
Telencephalon sham
Flare freqyency z-scored
n = 3
F
Telencephalon sham
G
rDm Lesion
Pre-lesion
Post-lesion
H
rDm Lesion
I
cDm Lesion
J
cDm Lesion
K
Telencephalon lesion
L
Telencephalon lesion

Figure S13: Coordination of flare response with animation following sham and lesion surgeries. **(A, C, E)** Average flaring frequency towards animation pre- and post-sham surgery associated with rDm **(A)**, cDm **(C)**, and telencephalon lesion **(E)**. **(B, D, F)** Correlation between flaring of sham-operated animals as controls of rDm **(B)**, cDm **(D)**, or telencephalon **(F)** lesions pre- and post-surgery with behaviors of the animation. **(G, I, K)** Average flaring frequency towards animation pre- and post-rDm **(G)**, cDm **(I)**, and telencephalon **(K)** lesion. **(H, J, L)** Correlation between flaring of rDm **(H)**, cDm **(J)**, and telencephalon **(L)** lesioned fish pre- and post-surgery with behaviors of the animation.

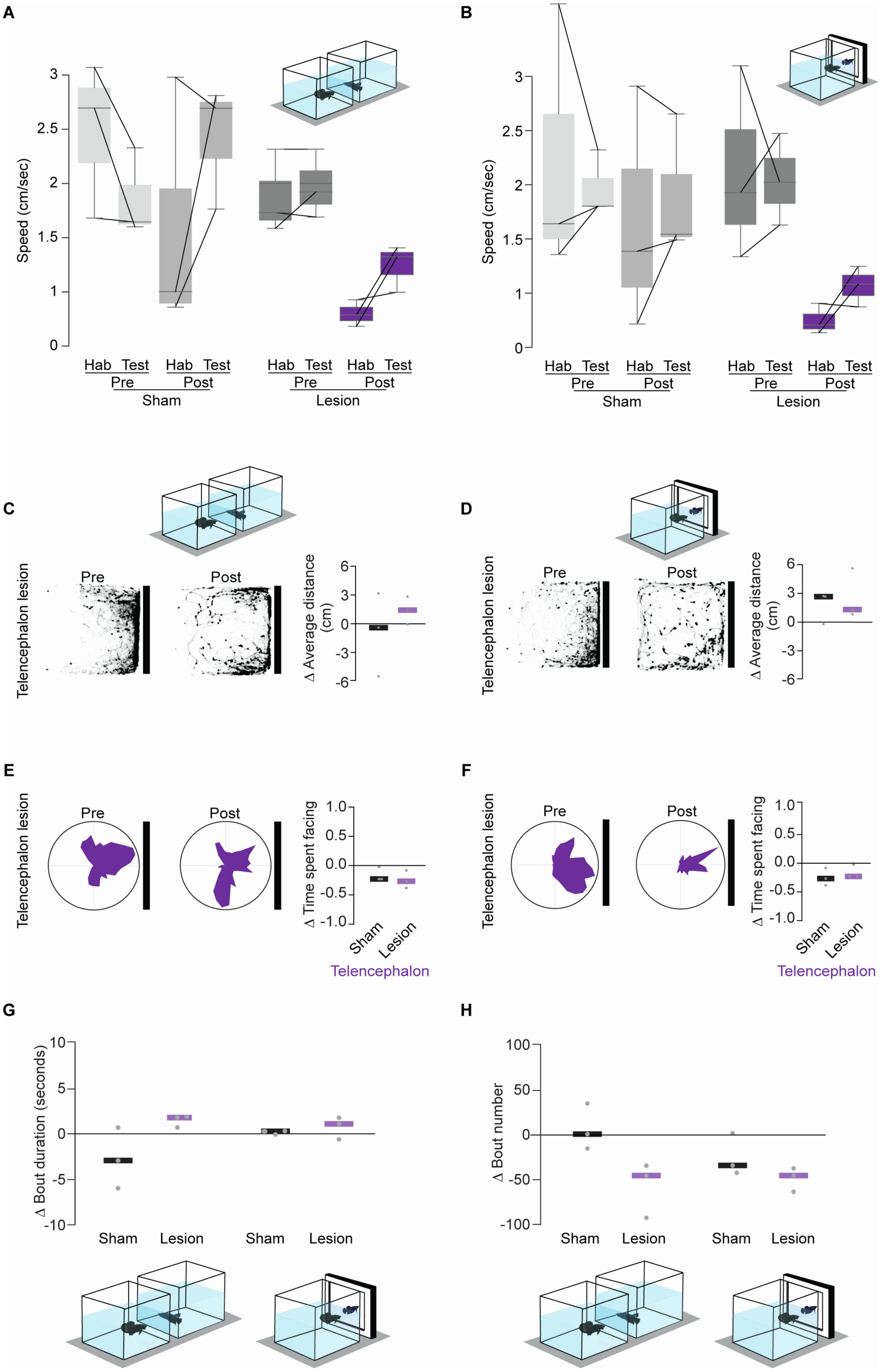
A
B
Speed (cm/sec)
0
0.5
1
1.5
2
2.5
3
Hab Test
Hab Test
Pre
Post
Sham
Lesion
C
D
Telencephalon lesion
Pre
Post
Δ Average distance (cm)
6
3
0
-3
-6
E
F
Δ Time spent facing
1.0
0.5
0.0
-0.5
-1.0
Sham
Lesion
Telencephalon
G
H
Δ Bout duration (seconds)
10
5
0
-5
-10
Δ Bout number
100
50
0
-50
-100
Sham
Lesion
Sham
Lesion

Figure S14: Additional metrics of behavior towards conspecific and animation following telencephalon lesion. (A) Average speed during free swimming before exposure to conspecific pre- and post- telencphalon lesion and associated sham controls. **(C, D)** Heatmaps showing tank occupancy (from a top view) by fish during conspecific **(C)** or animation **(D)** exposure pre- and post-lesion (left). Change in the distance to the conspecific **(C)** or animation **(D)** (right). **(E, F)** Polar plots showing distributions of the head orientation during conspecific **(E)** or animation **(F)** exposure pre- and post-lesion (left). Change (post-pre surgery) in the proportion time spent facing within 180º of the conspecific **(E)** or animation **(F)** (right). **(G)** Change (post-pre surgery) in flare bout duration against conspecific (left) or animation (right). **(H)** Change (post-pre surgery) in the number of flare bouts against conspecific (left) or animation (right). **(C-H)** Dots denote individuals with lines at the median.

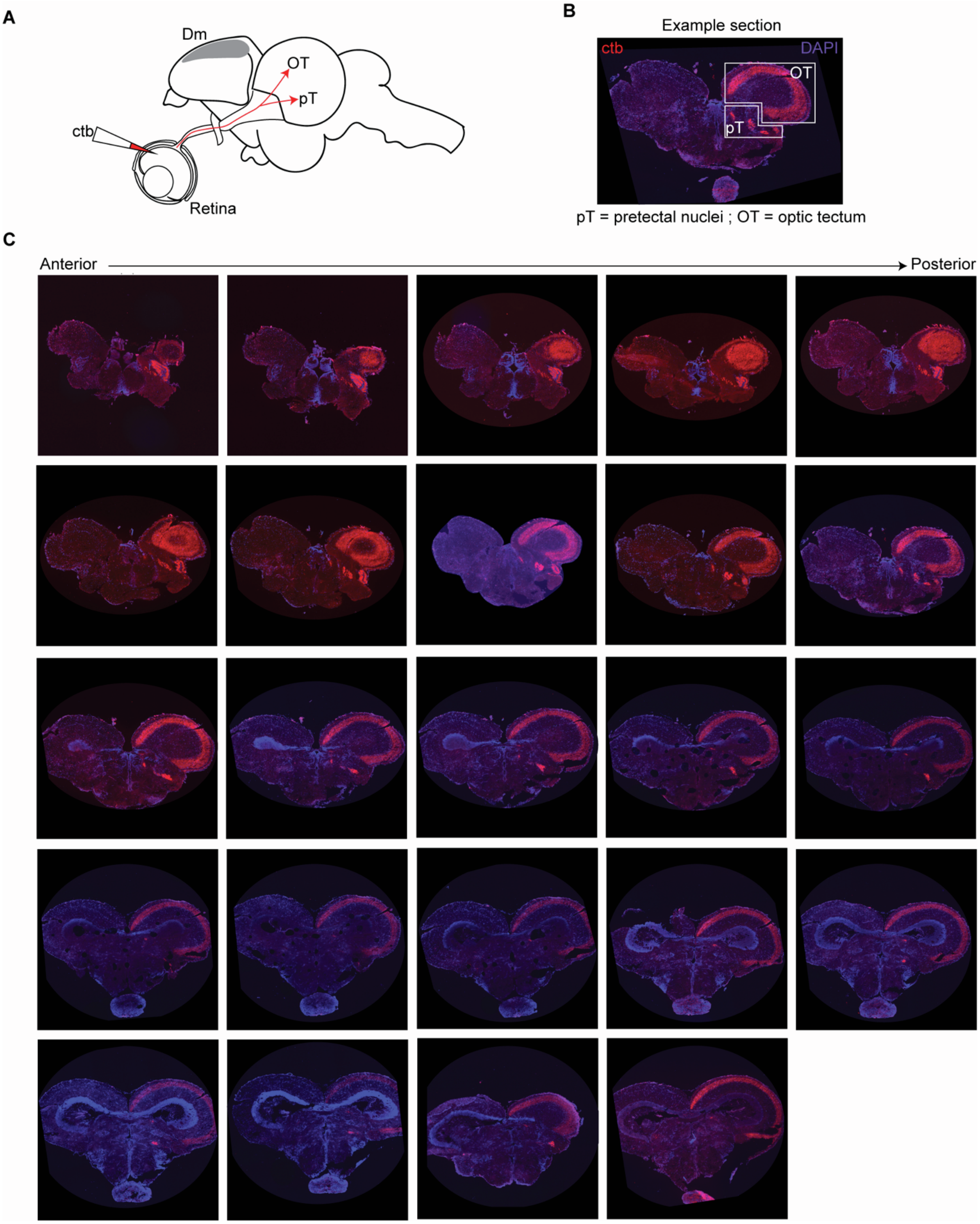

Figure S15: Retinorecipient nuclei in adult betta. (A) Schematic of injection site (intraocular) and presumptive retinal projections. **(B)** Labelled retinal ganglion cell (RGC) terminals in the optic tectum (OT) and a group of pretectal nuclei (pT). **(C)** RGC termination in OT and pT along anterior-posterior axis. Naming derived from Vigouroux et al. (ref. [5]). Three fish (2 females, 1 male) were included in the analysis.

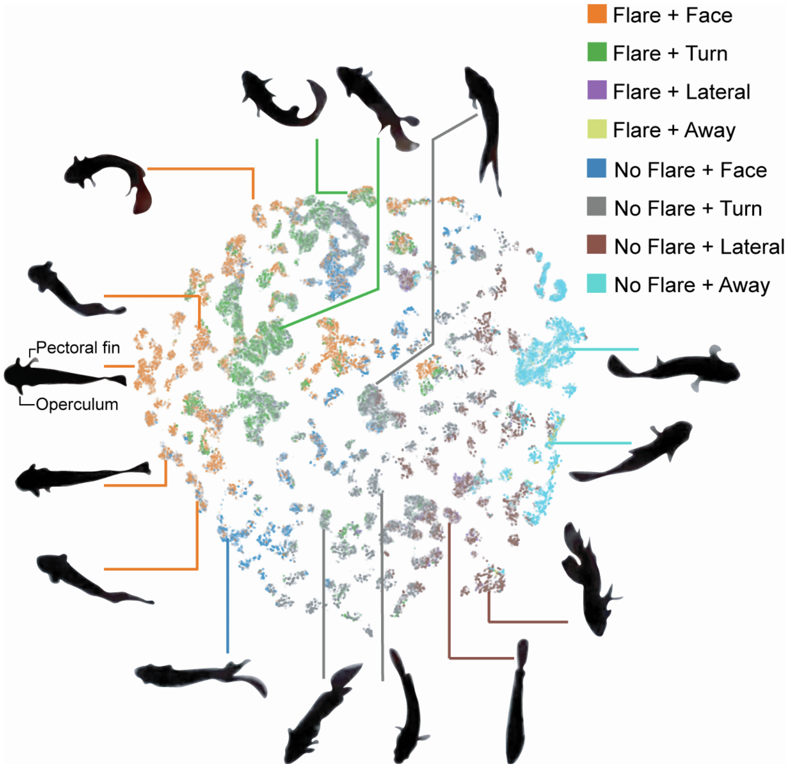

Figure S16: Unsupervised clustering of behavioral states during aggressive display. (**A**) tSNE of betta behavioral features (see Methods). Dots (corresponding to video frames) were colored by the manually-defined discrete states described in Figure S5. Around 50% of frames did not fall inside a cluster and are not plotted.

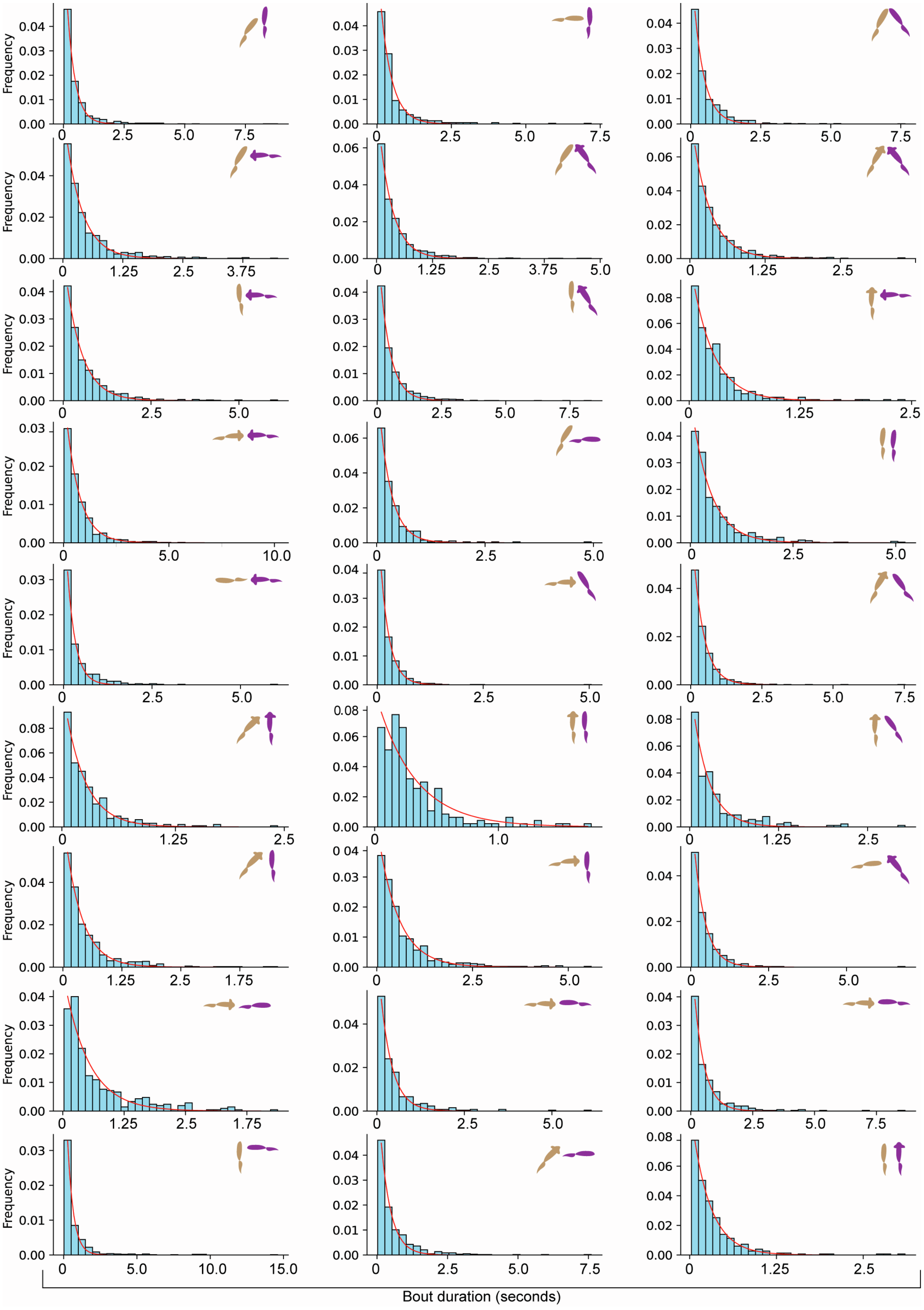
Frequency
Bout duration (seconds)

Figure S17: Frequency of durations of two-fish behavioral states. The durations of the two-fish states described in Figure S5 follow exponential distributions. Red denotes exponential fits.

Supplemental References

1. Magalhães Horn, Â.C., and Rasia-Filho, A.A. (2018). The cytoarchitecture of the telencephalon of *Betta splendens* Regan 1910 (*Perciformes: Anabantoidei)* with a stereological approach to the supracommissural and postcommissural nuclei. Anat. Rec. *301*, 88–110. https://doi.org/10.1002/ar.23699.

2. Marino-Neto J, S.RM. (1988). A stereotaxic atlas for the telencephalon of the Siamese fighting fish (*Betta splendens*). Braz J Med Biol Res *21*, 971–986.

3. Maruska, K.P., Butler, J.M., Field, K.E., and Porter, D.T. (2017). Localization of glutamatergic, GABAergic, and cholinergic neurons in the brain of the African cichlid fish, *Astatotilapia burtoni*. J. Comp. Neurol. *525*, 610–638. https://doi.org/10.1002/cne.24092.

4. Wullimann, M.F., Rupp, B., and Reichert, H. (1996). The brain of the zebrafish *Danio rerio*: a neuroanatomical atlas. In Neuroanatomy of the Zebrafish Brain: A Topological Atlas, M. F. Wullimann, B. Rupp, and H. Reichert, eds. (Birkhäuser), pp. 19–87. https://doi.org/10.1007/978-3-0348-8979-7_5.

5. Vigouroux, R.J., Duroure, K., Vougny, J., Albadri, S., Kozulin, P., Herrera, E., Nguyen-Ba-Charvet, K., Braasch, I., Suárez, R., Bene, F.D., et al. (2021). Bilateral visual projections exist in non-teleost bony fish and predate the emergence of tetrapods. Science *372*, 150–156. https://doi.org/10.1126/science.abe7790.
